# The applications of nanozymes in neurological diseases: From mechanism to design

**DOI:** 10.7150/thno.83370

**Published:** 2023-04-23

**Authors:** Yuan Zhang, Lei Zhang, Man Wang, Peifeng Li

**Affiliations:** Institute for Translational Medicine, The Affiliated Hospital of Qingdao University, Qingdao University, Qingdao, China.

**Keywords:** Nanozymes, neurological diseases, ROS

## Abstract

Nanozymes are a class of nanomaterials with enzyme-like catalytic activities. Due to their multiple catalytic activities, as well as their good stability, modifiable activity and other advantages over natural enzymes, they have a wide range of application prospects in sterilization, the treatment of inflammation, cancer, and neurological diseases, and other fields. In recent years, it has been found that various nanozymes have antioxidant activity, allowing them to simulate the endogenous antioxidant system and play an important role in cell protection. Therefore, nanozymes can be applied in the treatment of reactive oxygen species (ROS)-related neurological diseases. Another advantage of nanozymes is that they can be customized and modified in a variety of ways to increase their catalytic activity beyond that of classical enzymes. In addition, some nanozymes have unique properties, such as the ability to effectively penetrate the blood‒brain barrier (BBB) or to depolymerize or otherwise eliminate misfolded proteins, making them potentially useful therapeutic tools for the treatment of neurological diseases. Here, we review the catalytic mechanisms of antioxidant-like nanozymes, as well as the latest research progress and strategies for designing therapeutic nanozymes, aiming to promote the development of more effective nanozymes for the treatment of neurological diseases in the future.

## Introduction

Nanozymes are a class of nanomaterials with enzyme-like catalytic activities. In 2007, Gao et al. first found that Fe_3_O_4_ nanoparticles possessed catalytic activity similar to that of horseradish peroxidase (HRP) and that their catalytic mechanism and efficiency were similar to those of natural HRP 1. In 2013, Wang et al. demonstrated that nanozymes have catalytic activities similar to those of natural enzymes. These studies challenged the long-held belief that inorganic materials are biologically inert and initiated nanozyme research. With the development of the field, nanozymes came to be defined as nanomaterials that catalyze the conversion of enzyme substrates to products under physiologically relevant conditions and exhibit enzymatic kinetics, such as Michaelis‒Menten kinetics, even though the molecular mechanisms of the reactions may differ between nanozymes and the corresponding natural enzymes 2. Compared with natural enzymes, the preparation of nanozymes is characterized by a simple process, a low production cost, good stability and modifiable activity, giving nanozymes a wide range of application prospects in the fields of detection, sewage treatment, sterilization, and the treatment of inflammation, cancer, and neurological diseases. To date, hundreds of nanomaterials, including dozens of elements, have been reported to have enzyme-like activities. Among them, more than one hundred kinds of nanozymes have been used in disease treatment. Nanozymes can be divided into the following four categories according to their catalytic activities: oxidoreductases, hydrolases, isomerases, and synthases 3. Nanozymes with antioxidant-like activity have an important effect on reactive oxygen species (ROS) and reactive nitrogen species (RNS).

In vivo, reactive oxygen and nitrogen species (RONS) radicals are natural products of aerobic metabolism and play important roles in cell signaling and homeostasis 4. Different types of RONS can be interconverted with each other, and an increase in total RONS levels causes oxidative stress, which may induce cell damage. Various RONS, such as O_2_^·-^, H_2_O_2_, ∙OH, and ∙NO, are continuously produced via metabolic processes and play important roles in the regulation of physiological functions of the body 5. However, excessive ROS can cause oxidative stress in the body, eventually inducing a variety of types of irreversible damage 6. Therefore, removing excess ROS from the body is essential for maintaining normal physiological functions. Under normal physiological conditions, the intracellular antioxidant system removes excess ROS in a timely manner to maintain cell homeostasis 7. However, under some abnormal conditions, aberrantly increased levels of cellular ROS impair the antioxidant system, resulting in severe cell dyshomeostasis. Therefore, exogenous antioxidant enzymes are needed to resist oxidative stress damage caused by ROS.

In recent years, a variety of nanozymes have been found to have endogen-like antioxidant activities, such as superoxide dismutase (SOD), catalase (CAT) and glutathione peroxidase (GPx) activities, allowing them to breakdown ROS into molecules that are not highly reactive, such as O_2_ and H_2_O 8. Another advantage of nanozymes is that they can be modified in a variety of ways to increase their catalytic capability beyond that of classical enzymes. Therefore, nanozymes can mimic the endogenous antioxidant system and play an important role in cell protection. In addition, some nanozymes have unique properties, such as the ability to effectively penetrate the blood‒brain barrier (BBB), making them potentially useful therapeutic tools for the treatment of neurological diseases 9.

Here, we review the catalytic mechanisms of antioxidant-like nanozymes, as well as the latest research progress and strategies for designing therapeutic nanozymes, with the aim of promoting the development of more effective nanozymes for the treatment of neurological diseases in the future.

## Catalytic mechanisms of antioxidant-like nanozymes

Nanozymes are characterized by their catalytic activity. According to their catalytic functions, nanozymes can be classified as oxidoreductase-like, hydrolase-like, synthase-like and isomerase-like nanozymes. Among the four types of nanozymes, oxidoreductase-like nanozymes account for more than 90% of all nanozymes and mainly have oxidase (OXD)-like, peroxidase (POD)-like, SOD-like and CAT-like activities. The main function of antioxidant-like nanozymes, including SOD-, CAT- and GPx-like nanozymes, is to regulate the production or removal of ROS and RNS for the treatment of a variety of diseases related to oxidation‒reduction (redox) imbalance.

### SOD-like nanozymes

At present, more than 70 kinds of nanozymes have been found to have SOD-like activity, including CeO_2_ and some carbon materials such as fullerenes, in addition to Pt, Au, Cu, Mn, Ni, Co, Mo, Rh, Fe, V and other metals and oxides, carbides, nitrides and sulfides. SOD-like nanozymes catalyze the transformation of superoxide anion (O_2_^·-^) to O_2_ and H_2_O_2_, so they can remove excessive ROS and RNS in organisms and play an important role in preventing oxidative stress, which can protect cells, prevent aging and inhibit the inflammatory response 10. O_2_^·-^ is a highly oxidizing ROS that can trigger free radical collision reactions that produce more ROS and RNS. In reactions catalyzed by SOD, O_2_^·-^ can be transformed into H_2_O_2_ and O_2_, and the resulting H_2_O_2_ is further decomposed into H_2_O and O_2_ by CAT. Most SOD-like nanozymes also have CAT activity, and together, they can remove ROS more thoroughly, thus providing an advantage over natural SOD or other small antioxidant molecules. Therefore, SOD-like nanozymes play an important role in the treatment of inflammatory diseases, atherosclerosis, cardiovascular diseases such as ischemia‒reperfusion (I/R), and neurodegenerative diseases (NDDs).

**Figure 1:**
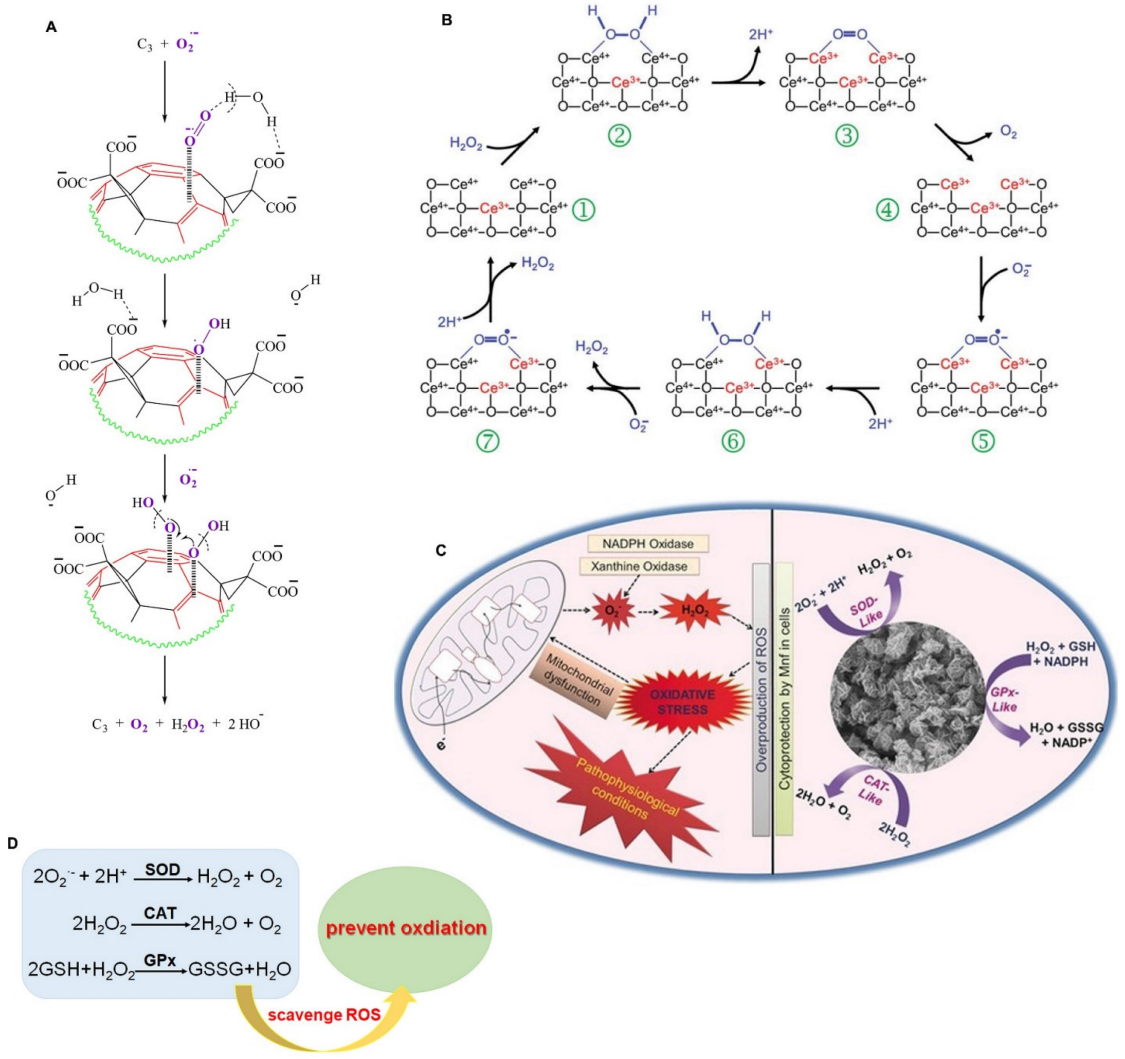
The schematic illustration of the catalytic mechanisms of antioxidant-like nanozymes. A. Schematic representation of SOD-like activity of C_3_ to scavenge O_2_^·-^. Adapted with permission from 12, copyright 2004, Elsevier. B. The catalytic mechanism of the CAT-like activity for CeO_2_ model. Adapted with permission from 16, copyright 2009, Royal Society of Chemistry. C. The catalytic mechanism of the GPx-like activity for Mn_3_O_4_ nanozyme. Adapted with permission from 27, copyright 2017, John Wiley and Sons. D. Nanozymes scavenge ROS to prevent oxidation by their redox reactions.

CeO_2_ was one of the first nanomaterials found to have SOD-like activity. The catalytic activity of CeO_2_ is mainly due to the directly convertible valence state and oxygen vacancy of Ce^3+^ and Ce^4+^, which can combine with superoxide and transfer electrons through redox reactions of H_2_O_2_, thereby reducing Ce^4+^ to Ce^3+^ 11. In addition, the SOD-like activity of many nanozymes depends mainly on the structure of the nanomaterials. Ali et al. reported that the electron-deficient region of fullerene C_60_ molecule (C_3_) nanozymes attracts O_2_^·-^ toward electron-deficient areas on its surface by electrostatic action, and then O_2_^·-^ is stabilized by hydrogen bonding with protons on the carboxyl groups (or intercalated solvating H_2_O) until the next O_2_^·-^ molecule combines with the original one, promoting the dismutation of O_2_^·-^ with the help of protons from the carboxyl groups and/or local water molecules (Figure 1A) 12. Gao et al. constructed carbon dot (C-dot) nanozymes with high SOD-like activity, comparable to that of the natural SOD enzyme 13. The SOD-like activity of C-dots depends on the hydroxyl and carboxyl groups binding superoxide anions, and carbonyl groups oxidizing superoxide anions to produce oxygen and reduced C-dots 13, 14. The reduced C-dots are oxidized back to their initial state by another superoxide anion and the reaction produces H_2_O_2_. Moreover, C-dot nanozymes can selectively target oxidatively damaged cells and mitochondria, significantly reduce intracellular ROS levels, and protect neurons from oxidative stress caused by ischemic stroke 13.

### CAT-like nanozymes

Nanozymes with CAT activity can catalyze the decomposition of H_2_O_2_ into H_2_O and O_2_, which can reduce the accumulation of ROS and effectively protect cells from oxidative stress damage. In addition, they can improve the O_2_-poor environment of tumors by generating O_2_, thus promoting cancer cell death 15. Since the discovery of the CAT-like activity of Fe_2_O_3_ nanoparticles, more than 100 kinds of CAT-like nanozymes have been discovered, including Au, Pt, Ag, Pd, Ir and other materials, as well as oxides, sulfides, and carbonitrides of some metals (such as Ce, Fe, Mn, Ru, Cu and Mo). The catalytic mechanism of CAT-like nanozymes is relatively simple and includes redox reactions and adsorption activation. The catalytic reaction of CAT-like nanozymes follows Michaelis‒Menten kinetics, and the catalytic capacity of these nanozymes increases with increasing substrate and mimic concentrations. For example, the CAT-like activity of CeO_2_, as a classical nanozyme, is influenced by the valence state of Ce in the redox reaction (Figure 1B) 11, 16. H_2_O_2_ binds to the Ce^4+^ site in CeO_2_, and the O-H bond in H_2_O_2_ breaks to release H_2_O. Then, the electrons are transferred from H_2_O_2_ to Ce^3+^, and Ce^4+^ is reduced to Ce^3+^ through a reduction reaction. Therefore, the high-valence state on the surface of CeO_2_ is beneficial to its CAT-like activity. The mechanism of adsorption activation is closely related to the catalytic mechanism of noble metal nanozymes. The OH* adsorbed on noble metal nanozymes (* represents adsorption on the metal surface) can promote the decomposition of H_2_O_2_ to produce H, and the adsorbed OH* can react with the generated H to produce HO_2_* and H_2_O*. Then, the generated HO_2_* passes H to another H_2_O_2_, leaving O_2_* and subsequently converting H_2_O_2_* to H_2_O* and OH* 17.

Zhang et al. prepared a defective Fe-N4 nanozyme via edge-site engineering (Fe-SANzyme) and achieved excellent CAT-like activity. During the preparation of the nanozymes, iron(II) phthalocyanine (FePc) molecules were loaded into the cavities of the zeolitic imidazole framework (ZIF-8) to reconstruct the frame structure. High-temperature carbonization of this frame structure can further introduce a large number of layered porous defects and produce abundant edge-hosted defective Fe-N4 atomic sites. The defects introduce notable charge transfer from the Fe atom to the carbon matrix, further activating the central Fe to strengthen the interaction with H_2_O_2_ and weaken the O-O bond. Thus, Fe-SANzyme can significantly remove ROS and relieve oxidative stress through CAT-like catalysis 18.

### GPx-like nanozymes

GPx is a member of the protease superfamily. It is the first selenoenzyme found in mammals and is an important selenophenase in the body 19, 20. GPx is an important member of the antioxidant system and has 8 subtypes, which can metabolize intracellular ROS and help maintain cell homeostasis. Studies have shown that selenium is an essential component of the catalytic reaction of GPx. Selenium, in the form of selenocysteine (Sec), catalyzes the decomposition of hydroperoxides by GSH in vivo, thus preventing peroxidation damage to cell membranes and other biological tissues 21, 22. Therefore, the main catalytic process of GPx-like nanozymes is the catalytic reduction of H_2_O_2_/organic hydroperoxide to H_2_O/alcohol under reduced GSH 23. To date, studies have shown that GPx-like nanozymes have two catalytic mechanisms, that is, ping-pong and ordered mechanisms 24, 25. Huang et al. constructed a graphene oxide-based selenium (GOSe) nanozyme with excellent GPx-like activity to protect cells from oxidative stress. The GOSe nanozyme reacts with H_2_O_2_ to obtain a selenium oxide intermediate. Then, this intermediate returns to its original state by oxidizing GSH to GSSG 26. In addition, Mn_3_O_4_ and vanadium also possess GPx mimic activity. Singh et al. found that Mn_3_O_4_ nanozymes exhibit GPx-, SOD- and CAT-like activities that protect cells from MPP+-induced cytotoxicity. In addition, they found that the activity of GPx was closely related to the valence ratio; that is, Mn_3_O_4_ with a higher Mn^3+^/Mn^2+^ ratio had higher GPx-like activity (Figure 1C) 27. Vernekar et al. found that V_2_O_5_ nanowires (Vn) exhibit excellent GPx-like activity. V_2_O_5_ inhibits intracellular oxidative stress by catalyzing the reduction of H_2_O_2_ in the presence of GSH 28. They also found that the size, morphology and surface-exposed crystal facets of the nanomaterials can affect the GPx-like activity of V_2_O_5_ 29.

In conclusion, the active components, charge state, interfacial structure and other factors determine the types of SOD-, CAT- and GPx-like activities of nanozymes and their catalytic capacity (Figure 1D). Clarifying the relationship between geometric structure and catalytic activity can help to improve the antioxidant activity of nanozymes so that they can be applied in the treatment of neurological diseases.

## The potential applications of nanozymes in neurological diseases

Oxidative stress and inflammation induced by oxidative damage are believed to be key factors in the occurrence and development of various neurological diseases. Many nanozymes have been found to be effective in protecting neuronal cells from oxidative injury and thus can prevent and cure some neurological diseases. Furthermore, nanozymes have the advantages of a stable structure, modifiable activity and diverse functions, giving them high application potential in the treatment of ROS-related neurological diseases.

### The potential applications of nanozymes in ischemic stroke

Stroke is a worldwide public health threat that is the second leading cause of death and a leading cause of disability. Ischemic stroke, which is mainly caused by the blockage of blood vessels that supply blood to brain tissue, results in brain tissue ischemia and a series of pathophysiological changes and accounts for approximately 87% of stroke cases 30. Reperfusion therapy involving intravenous thrombolysis and intravascular thrombectomy is currently recognized as an effective treatment. However, when blood flow is restored during reperfusion therapy, a large amount of ROS is produced, leading to cerebral I/R injury, which aggravates neurological impairment. Therefore, antioxidant therapy, which promotes ROS clearance, has also been developed as a treatment strategy for ischemic stroke 31, 32. However, exogenous supplementation with natural antioxidant enzymes has some disadvantages. For example, there are limited sources of these enzymes, they have poor stability in vivo, and they are expensive. Therefore, efficient, stable and safe antioxidant enzyme mimics are urgently needed. Many nanozymes have been found to be effective in protecting nerve cells from oxidative damage and can be used to alleviate neurological diseases 31, 32. Nanozymes overcome many of the disadvantages of traditional drugs used to treat ischemic stroke, as modified nanozymes are not rapidly decomposed in systemic circulation, leading to a prolonged half-life, improved stability and the ability to cross the BBB. Most importantly, nanozymes have multiple potent antioxidant enzyme activities, and they can significantly inhibit the production of a large number of RONS during cerebral I/R injury, prevent oxidative stress to effectively ameliorate brain injury and promote the recovery of nerve function after stroke (Table 1).

For example, Xi et al. introduced Fe into the structure of nitrogen-doped carbon nanozymes to construct peroxisome-like nanozymes (Fe-N4 nanozymes). Nanozymes have outstanding SOD, CAT, POD, OXD, and uricase (UOD) activities and are capable of regulating uric acid and ROS levels, thereby ameliorating hyperuricemia and ischemic stroke 33. Huang et al. showed that HSA-Mn_3_O_4_ can effectively inhibit brain tissue damage by restraining cell apoptosis and endoplasmic reticulum (ER) stress in ischemic stroke models. Mn_3_O_4_ is an inorganic nanomaterial containing manganese that can release manganese (Mn) ions into circulation and promote the activity of superoxide dismutase 2 (SOD_2_) in the body. Human serum albumin (HSA) can increase the stability of Mn_3_O_4_ in the circulation and effectively reduce inflammation in vivo. Studies have shown that HSA-Mn_3_O_4_ can effectively release Mn ions and can act as the metal core of SOD_2_ to promote its expression and activity. Therefore, HSA-Mn_3_O_4_ can inhibit the production of the free radical ·OH by neurons after I/R while effectively alleviating ER stress and reducing oxidative stress levels in cells, playing a dual neuroprotective role and ultimately ameliorating reperfusion injury to treat ischemic stroke 34. Huang et al. prepared a Ce/Zeo-NMs nanozyme with SOD and CAT catalytic activities and found that it can reduce intracellular Zn^2+^ and ROS levels in the ischemic area, thereby protecting against brain damage and improving neurological performance. Ce/Zeo-NMs alleviated BBB disruption by inhibiting the degradation of tight junction proteins (TJPs) and decreasing the infarct volume caused by I/R injury in a middle cerebral artery occlusion reperfusion (MCAO/R) model. Furthermore, Ce/Zeo-NMs can inhibit the activation of microglia and astrocytes and the release of proinflammatory cytokines, such as tumor necrosis factor-α (TNF-α) and interleukin 6 (IL-6), exerting neuroprotective effects for the treatment of ischemic stroke 35. Yan et al. reported that a PEG-modified Fe_3_O_4_ nanozyme with SOD-like activity effectively reduced the cerebral infarct volume and neuronal death in a mouse model after cerebral ischemic stroke. PEG-modified Fe_3_O_4_ nanozymes can diffuse into the cerebral vasculature, normalize the local redox state, decrease blood malondialdehyde (MDA) levels and increase Cu/Zn SOD levels. Furthermore, Fe_3_O_4_ nanozymes increase the levels of platelet endothelial cell adhesion molecule-1 (PECAM-1), Zonula Occluden (ZO)-1 and Claudin-5, which play important roles in restoring BBB integrity, in the hippocampus of cerebral ischemic stroke mice 36. In another study, Liu et al. developed a Co-Fe_3_O_4_ nanozyme that exhibited CAT-like activity and was capable of scavenging H_2_O_2_, O_2_^·-^, ·NO, and ONOO-. They demonstrated that the Co-Fe_3_O_4_ nanozyme could decrease intracellular ROS levels and inflammatory factor levels to confer neuroprotection against ischemic stroke. In addition, Co-Fe_3_O_4_ was found to accumulate specifically in the infarct rim and to be endocytosed by neurons, astrocytes, microglia, and endothelial cells to reduce the infarct volume in stroke models 37. Tian et al. constructed Fe_2_NC@selenium (Fe_2_NC@Se) nanozymes with enhanced SOD-like, CAT-like, and GPx-like activities, which exerted a protective effect against cerebral I/R injury. Fe_2_NC@Se treatment significantly decreased intracellular ROS levels and apoptosis in oxygen and glucose deprivation/reoxygenation (OGD/R)-exposed cells. Furthermore, the researchers reported that Fe_2_NC@Se treatment could decrease the brain infarct volume and edema and reduce oxidative stress-induced brain injury in MCAO rats. Fe_2_NC@Se improved neurological function and suppressed neural apoptosis by inhibiting the ASK1/JNK signaling pathway 38. Feng et al. reported that a neutrophil-like cell membrane-coated mesoporous Prussian blue nanozyme (MPBzyme@NCM) can be delivered to damaged brain tissue after stroke due to the innate connection between microvascular endothelial cells and neutrophils in the brain. Thus, this nanozyme represents a noninvasive targeted therapy for ischemic stroke. Although the SOD and CAT activities of MPBzyme@NCM are weaker than those of natural enzymes, treatment with MPBzyme@NCM inhibited microglial M1 polarization, facilitated M2 polarization, reduced the recruitment of neutrophils, promoted neurogenesis and protected against neuronal damage after stroke 39. Wang et al. constructed a peptide-based MnO_2_ nanozyme (PNzyme/MnO_2_) to bind a thrombolytic polypeptide and a stroke homing peptide, thereby effectively treating ischemic stroke. PNzyme/MnO_2_ has SOD- and CAT-like activities that eliminate ROS produced during I/R. Multifunctional PNzyme/MnO_2_ can cross the BBB and accumulate in ischemic tissues by binding to the transferrin receptor T7 sequence (HAIYPRH) on endothelial cells and the stroke homing sequence (CLEVSRKNC) of apoptotic neurons in ischemic stroke tissues. The PNzyme/MnO_2_ nanozyme specifically binds to ischemic thrombus via the fibrin-binding motif (CREKA). Thrombin recognizes and cleaves the LTPRGWRLGGC sequence, releasing a thrombolytic peptide (GRPAK) that initiates the thrombolytic process. The ROS produced after thrombolysis and reperfusion can be effectively eliminated by the catalytic activity of a cascade of SOD-CAT-like nanozymes, resulting in a strong thrombolytic effect and reducing ischemic injury to the brain tissue 40.

Overall, nanozymes play an important role in the treatment of ischemic stroke by eliminating ROS through antioxidant effects, thus inhibiting the inflammatory response, alleviating brain damage caused by oxidative stress, and decreasing cerebral infarct volume and edema.

### The potential applications of nanozymes in NDDs

NDDs, which are characterized by delayed onset and selective neuronal dysfunction, are a class of irreversible neurological diseases caused by the loss of neurons in the brain and spinal cord; NDDs include Parkinson's disease (PD), Alzheimer's disease (AD), Huntington's disease (HD), etc. The pathological mechanisms of NDDs are closely related to misfolded protein accumulation, oxidative stress, the inflammatory response, autophagy and metal ion disorder. In recent years, increasing data on the therapeutic efficacy of nanozymes for NDDs have been reported (Table 1). Nanozymes not only have SOD-like, CAT-like and GPx-like enzyme activities, which allow them to remove ROS but also proteinase-like activities, which allow them to remove accumulated misfolded proteins, such as amyloid-beta (Aβ) aggregates. In addition, some nanozymes can also act as metal-chelating agents to remove copper and iron ions, thus playing an important role in maintaining metal ion homeostasis in the brain and removing intracellular excess ROS produced by metal ions.

PD is the second most common neurological disease in elderly individuals. The most important pathological changes associated with PD are the death of dopaminergic neurons in the midbrain substantia nigra, a significant reduction in dopamine content in the striatum, and the aggregation of alpha-synuclein (ɑ-syn) into Lewy bodies in the cytoplasm of remaining nigral neurons. The pathogenesis of PD involves many processes, including autophagy in the substantia nigra, mitochondrial dysfunction, the inflammatory response and oxidative stress. In recent years, it has been found that nanozymes can effectively alleviate neuronal injury in PD by reducing ROS levels, protecting mitochondria from oxidative damage and protecting dopaminergic neurons against neuroinflammation. For example, Singh et al. synthesized Mn_3_O_4_ nanoflowers (Mnfs) with SOD-, CAT- and GPx-like activities and found that they reduced ROS levels and the activation of caspase-3/7 in a 1-methyl-4-phenylpyridinium (MPP+)-induced PD-like cellular model, indicating that they may protect against neurological disorders such as PD 27. Ma et al. constructed a Prussian blue nanozyme (PBzyme) that showed good SOD- and CAT-like activities and exhibited excellent ROS-scavenging capability in a 1-methyl-4-phenyl-1,2,3,6-tetrahydropyridine (MPTP)-induced PD mouse model. A study showed that PBzyme is a pyrogenic inhibitor that alleviates neurodegeneration in PD mice and protects microglia and neurons from MPTP-induced toxicity. PBzyme can alleviate motor deficits, reduce mitochondrial membrane damage, and rescue dopaminergic neurons. In MPTP-induced PD model mice, the intraventricular administration of PBzyme reduced dopaminergic degeneration and inhibited neuroinflammation. In vitro and in vivo experiments showed that PBzyme can reduce the activation of the microglial nucleotide-binding domain, the leucine-rich repeat family pyrin domain containing 3 (NLRP3) inflammasome, and caspase-1 by scavenging ROS, thereby decreasing gasdermin D (GSDMD) cleavage, as well as inflammatory factor production, and eventually leading to the inhibition of microglial pyroptosis. Thus, PBzyme exerts a neuroprotective effect as a pyrogenic inhibitor and represents a new agent for the treatment of PD 41. Li et al. developed a lactoferrin (Lf)-modified Au-Bi2Se3 nanodot (Lf-Au-Bi_2_Se_3_) that efficiently alleviates PD. The Lf-Au-Bi_2_Se_3_ nanozyme exhibited SOD-, CAT-, and GPx-like activities, allowing it to scavenge ROS, as well as a good ability to cross the BBB. Intravenous injection of Lf-Au-Bi_2_Se_3_ can significantly protect mitochondria from oxidative stress and suppress dopaminergic neuron loss in the substantia nigra pars compacta, which implied that it could efficiently protect dopaminergic neurons in the MPTP-induced PD mouse model 42. Feng et al. constructed a two-dimensional (2D) vanadium carbide (V_2_C) MXene nanoenzyme (2D V_2_C MXenzyme) with SOD-, CAT-, POD-, GPx-, thiol peroxidase (TPx)- and haloperoxidase (HPO)-like activities and found that it markedly reduced ROS levels. A study showed that the 2D V_2_C MXenzyme can effectively protect against ROS-related diseases, such as PD. Treatment with V_2_C MXenzyme can help maintain tyrosine hydroxylase (TH) activity and effectively ameliorate neuroinflammation in PD mice, indicating that V_2_C MXenzyme treatment significantly protects mice from neurotoxicity by inhibiting MPTP-induced oxidative stress 43. Ji et al. developed self-catalytic small interfering RNA (siRNA) nanocarriers (S/Ce-PABMS) with ceria (CeO_2_)-like activity to carry siRNA targeting SNCA (siSNCA) and found that they reduced α-syn aggregation. S/Ce-PABMS not only has good ROS scavenging ability but can also effectively penetrate the BBB because of its action as an acetylcholine analog. In vivo experiments have shown that S/Ce-PABMS treatment can inhibit microglial activation, repair dopaminergic neurons and regulate the release of inflammatory cytokines, thereby alleviating neuroinflammation and significantly improving the spontaneous motor ability and coordination of PD mice, indicating that these nanozymes may be effective in treating PD 44.

AD is the most common neurodegenerative disease in the elderly population and is characterized by memory loss and cognitive impairment. The pathological features of AD include the aggregation of misfolded Aβ protein in the extracellular space in the brain, the formation of tau neurofibrillary tangles, elevation of ROS levels and metal ion homeostasis disorder. Nanozymes can inhibit oxidative stress induced by Aβ aggregation by reducing intracellular ROS levels and they have protease-like activities, allowing them to clear Aβ aggregates. For example, Jia et al. constructed a borneol (Bor)-modified octahedral palladium (Pd@PEG@Bor) nanozyme with antioxidant activity to eliminate ·OH and found that it markedly decreased the number of Aβ plaques in an AD model. Pd@PEG@Bor can eliminate intracellular ROS and rescue Ca^2+^ homeostasis, and it has good BBB penetration. When administered intravenously, Pd@PEG@Bor can inhibit the production and aggregation of Aβ, reduce neuroinflammation, and further ameliorate cognitive impairment in AD mice, indicating that it is a potential therapeutic agent for AD 45. Xia et al. reported that PEG-Fe_3_O_4_ nanozymes can protect against brain aging by improving neuroblast differentiation in the hippocampal dentate gyrus. PEG-Fe_3_O_4_ nanozymes have SOD- and CAT-like activities, allowing them to scavenge ROS and thus markedly reduce apoptosis and BBB injury induced by D-galactose treatment. Long-term treatment with PEG-Fe_3_O_4_ nanozymes can improve BBB integrity by rescuing the reductions in PECAM-1, claudin-5 and ZO-1 protein expression in the hippocampal dentate gyrus in D-galactose-treated aged mice, as well as by inhibiting autophagy via inactivation of the Akt/mTOR signaling pathway, indicating that PEG-Fe_3_O_4_ nanozymes have a potential neuroprotective effect in AD 46. Ren et al. designed (3-carboxypropyl) triphenyl-phosphonium bromide-conjugated 1,2-distearoyl-sn-glycero-3-phosphoethanolamine-N-[amino(polyethylene glycol)-2000]-functionalized molybdenum disulfide quantum dots (TPP-MoS_2_ QDs) that target mitochondria, and these nanozymes represent a new therapeutic agent for AD. A study showed that TPP-MoS_2_ QDs possess SOD- and CAT-like activities, allowing them to scavenge ROS, cross the BBB, and escape from lysosomes to target mitochondria; these activities help prevent spontaneous neuroinflammation by regulating the levels of the proinflammatory substances interleukin 1β (IL-1β), IL-6, TNF and anti-inflammatory transforming growth factor β (TGF-β). Furthermore, TPP-MoS2 QDs were found to alleviate Aβ aggregate-induced neurotoxicity by converting microglia from the proinflammatory M1 phenotype to the anti-inflammatory M2 phenotype and had the ability to ameliorate Aβ deposition and neuronal loss in the hippocampus of AD mice 47. Ma et al. reported that amyloid-targeting, N-doped three-dimensional mesoporous carbon nanospheres (KD8@N-MCNs) can degrade Aβ aggregates and are accordingly potential therapeutic agents for the treatment of AD. KD8@N-MCNs have SOD-like and CAT-like activities, and they remove excess ROS in cells and reduce neuroinflammation in AD. In addition, KD8@N-MCNs effectively cross the BBB and dissociate Aβ aggregates due to the Lys-Leu-Val-Phe-Phe (KLVFF) target peptide covalently grafted to the surface of the nanospheres. In vivo studies have shown that KD8@N-MCNs reduce Aβ deposition, alleviate memory deficits and reduce neuroinflammation in 3xTg-AD mice. Furthermore, KD8@N-MCNs have excellent photothermal effects and stability and can degrade Aβ aggregates in response to NIR-II radiation, which can penetrate through the dense scalp and skull, showing that KD8@N-MCNs have the potential to be used for photothermal treatment of AD 48. In addition, Ma et al. constructed a bioinspired antioxidant nanozyme coated with an Aβ-targeting peptide-modified erythrocyte membrane (denoted Cu_x_O@EM-K) that can specifically clear peripheral Aβ. Cu_x_O@EM-K is made of Cu_x_O wrapped by a modified 3xTg-AD mouse erythrocyte membrane with the Aβ-targeting peptide KLVFF. The study showed that Cu_x_O@EM-K possesses excellent SOD- and CAT-like activities, allowing it to reduce ROS production induced by Aβ. Long-term injection of Cu_x_O@EM-K can effectively reduce brain Aβ burden and rescue memory deficits in 3xTg-AD mice 49. Du et al. constructed a 2D niobium carbide (Nb_2_C) MXene-based nanozyme (Nb_2_C MXenzyme) that possesses SOD and CAT activity, which allows it to scavenge ROS and capture superfluous Cu^2+^. Furthermore, Nb_2_C MXenzyme was determined to have the capability to cross the BBB and effectively suppress Aβ aggregation. Nb_2_C MXenzyme treatment can alleviate mitochondrial and neuroglial damage, suppress neuroinflammation, and ameliorate cognitive deficits in APP/PS1 double transgenic mice 50. Ge et al. designed KLVFF@Au-CeO_2_ nanozymes, in which the KLVFF peptide is adsorbed on the middle surface of gold nanorods (Au NRs), and both ends of the Au NRs are coated with CeO_2_ NPs. Therefore, KLVFF@Au-CeO_2_ nanozymes present CAT- and SOD-like activities. The addition of hot electrons produced by a plasma photothermal effect can expand the area in which CeO_2_ exerts photocatalytic activity to the near-infrared region (NIR), significantly improving its redox performance. Moreover, modification of the Aβ-targeted inhibitory peptide KLVFF significantly improved the BBB-penetrating ability. In vivo experiments demonstrated that KLVFF@Au-CeO_2_ nanozymes improved the cognitive function of AD mice by inhibiting the aggregation of Aβ monomers, promoting the depolymerization of Aβ fibers, and scavenging ROS 51.

In addition, nanozymes have been reported to be useful for the treatment of HD. HD is an autosomal dominant hereditary NDD. The pathological mechanism of HD is the abnormal expansion of CAG in the gene encoding the huntingtin (HTT) protein, leading to the formation of mutant HTT (mHTT). The aggregation of neurotoxic mHTT in the brain affects neuronal activity in the caudate nucleus and medium spiny neurons in the putamen and thus causes motor, cognitive and psychiatric dysfunction in patients 52. A boehmite amino-nanozyme (BNP) with therapeutic effects against HD was developed. BNP nanoparticles have SOD-like activity, allowing them to scavenge Cu^2+^ and mitochondrial reactive oxygen species (mitoROS) and disaggregate mutant HTT deposits in cells, indicating that BNP has the potential to affect HD 53.

Nanozymes can delay the progression of NDDs by regulating ROS levels, suppressing misfolded protein aggregation and helping maintain metal ion homeostasis in cells, indicating that nanozyme administration may be a potential therapeutic strategy for NDDs.

### The potential applications of nanozymes in traumatic brain injury

Traumatic brain injury (TBI) is brain tissue damage caused by an external blow or shock. TBI presents in various forms, ranging from mild alterations of consciousness to an unrelenting comatose state and death. In severe TBI, the entire brain is affected by a diffuse injury and swells. The pathological mechanism of TBI is complex, and TBI is caused by external physical factors or secondary injury. TBI, as one of the most serious brain injuries, can trigger a series of biochemical reactions and neuroinflammation in the brain and consequently induce irreversible nerve tissue damage. A large number of RONS are produced during TBI. Moreover, excessive free radicals spread into neuronal cells, where they promote lipid peroxidation, protein carbonylation and DNA oxidation, cause abnormal changes in membrane permeability and fluidity and further induce cell apoptosis, necrosis and autophagy, eventually leading to cell death and causing severe neurotoxicity. Therefore, free radical scavenging has long been a feasible therapeutic strategy for TBI. Studies have shown that a variety of nanozymes can remove RONS and may therefore be effective therapies for TBI (Table 1).

For example, Zhang's group constructed a carbogenic nanozyme with high selectivity for reactive nitrogen radicals and the capability of scavenging RNS, such as ·NO and ONOO-, as well as traditional reactive oxygen radicals, such as H_2_O_2_ and ·OH. In vitro experiments have shown that carbogenic nanozymes can rescue neuronal cells damaged by hydrogen peroxide or lipopolysaccharide by removing RONS. In addition, based on their multiple enzyme activities, carbogenic nanozymes can eliminate harmful hydrogen peroxide and glutathione disulfide bonds and efficiently improve the spatial memory of TBI mice, indicating that they have the potential to treat acute brain trauma 54. Zhang's group also designed a PtPdMo trimetallic (PtPdMo triM) nanozyme with high catalytic activity and environmental selectivity. The PtPdMo trim nanozyme has multiple enzyme activities, including SOD- and CAT-like activities, that provide the ability to scavenge ROS and RNS. In vitro experiments have shown that triM nanozymes can improve the survival rate of injured nerve cells. SOD activity and lipid peroxidation were significantly restored in an LPS-induced brain injury model after triM nanozyme treatment. In addition, triM nanozyme treatment significantly improved the survival rate of mice with brain injury, alleviated neuroinflammation and improved memory 55. Subsequently, the researchers constructed and synthesized a Pt/CeO_2_ nanozyme-based bandage with lasting catalytic activity for the noninvasive treatment of TBI. The synthesis of Pt/CeO_2_ is based on the principle of single-atom catalysis. The distribution of single-atom Pt leads to the lattice expansion of CeO_2_, allowing the formation of a new stable active site and electron transfer path and leading to a significant increase in catalytic capacity. Pt/CeO_2_ exhibits excellent SOD-, CAT-, and GPx-like activities, as well as the capability of scavenging RONS. Treatment with Pt/CeO_2_ nanozymes can significantly improve wound healing after neurotrauma and reduce neuroinflammation in mice with brain trauma 56. Zhang's group also constructed another nanozyme-based bandage loaded with Cr-doped CeO_2_ (Cr/CeO_2_) nanozymes by single-atom catalysis, and the bandage exhibited multiple enzyme activities, such as SOD-, CAT- and GPx-like activities, and scavenged RONS. Furthermore, Cr/CeO_2_ nanozymes can protect neuronal cells against oxidative damage induced by LPS by decreasing excessive RONS production. Treatment with Cr/CeO_2_ nanozymes can promote wound healing, reduce neuroinflammation and improve cognition in mice following brain trauma 57. Zhang et al. also reported an oligomeric nanozyme (O-NZ) with ultrafast electron transfer and ultrahigh catalytic activity. O-NZ shows excellent SOD- and GPx-like activities and can rapidly remove O_2_^·-^, ·NO and ONOO. O-NZ nanozyme treatment significantly increased the overall survival rate and greatly improved the neurocognition and memory of mice with severe TBI, and it reduced neuroinflammation by regulating the nuclear factor erythroid-2 related factor 2 (Nrf2)/heme oxygenase-1 (HO-1) pathway 58. Recently, Zhang's group constructed RhN_4_, VN_4_ and Fe-Cu-N_6_ nanozymes with ultrahigh biological activity, and their affinity for biocatalysis was found to be 5-20 times that of natural enzymes. RhN_4_ and VN_4_ possess high peroxidase activity, with an affinity 4-5 times that of natural enzymes. RhN_4_ also has extremely high CAT-like activity, showing 20 times higher activity than CAT itself. VN_4_ exhibits excellent GPx-like activity, showing 7 times higher activity than GPx itself. The evidence shows that the single-atom nanozymes RhN_4_, VN_4_ and Fe-Cu-N_6_ can be used to promote efficient wound healing. Single-atom nanozymes possess various oxidoreductase activities. By promoting the secretion of vascular endothelial growth factor, they regulate the oxidative stress response, reduce wound inflammation, accelerate the transition of wound closure from inflammation to tissue repair, and promote efficient healing of the scalp after brain trauma. Furthermore, they regulate macrophages and other immune cells, inhibit the activation of microglial cells, and alleviate oxidative damage after brain trauma 59.

The antioxidant effects of nanozymes can effectively eliminate excess ROS and RNS to regulate REDOX homeostasis. Therefore, the antioxidant properties of nanozymes can alleviate oxidative damage and play an increasingly important role in the treatment of brain trauma.

## Strategies for designing nanozymes for the treatment of neurological diseases

Nanozymes are nanomaterials with enzyme-like properties. The material composing the nanozyme affects its properties, including its chemical composition, synthesis method, and form. Nanozymes exhibit the catalytic activities of POD-, CAT-, SOD-, glucose oxidase (GOx)- and GPx-like enzymes. In addition, nanozymes need to be able to effectively penetrate the BBB, depolymerize or remove misfolded proteins, and inhibit inflammatory responses to effectively treat neurological diseases.

### Improvement of antioxidative ability

Oxidative damage and inflammation caused by oxidative stress are thought to be major etiological factors of brain injury, aging, and chronic neurodegeneration. Cellular oxidative stress is caused by abnormally elevated ROS levels and leads to DNA damage, inflammatory responses, cell senescence, and programmed cell death. Peroxides and nitrogen oxides mediate oxidative damage through a number of independent mechanisms, including lipid peroxidation, DNA damage, and activation of anti-poly (ADP-ribose) antibodies and polyadenosine diphosphate ribose polymerase (PARP), to produce cytotoxic effects. Free radicals may react with all cellular macromolecules, resulting in liposomal peroxidation and the oxidation of DNA and proteins. Liposomal peroxidation may cause membrane damage, and the end products of liposomal peroxidation, such as 4-hydroxynonenal (4-HNE), are toxic to neurons and the white matter of the brain, including axons and oligodendrocytes, and can cause cell death 60. Damage to proteins, especially enzymes, can impair their function. DNA peroxidation activates repair enzymes, such as PARP, which causes rapid depletion of intracellular energy and leads to cell death. In normal cells, many antioxidant enzymes clear excess ROS and maintain mitochondrial function 61. In diseased cells, however, the levels of these enzymes are insufficient to remove excess ROS, or the enzymes may even be dysfunctional. Therefore, exogenous antioxidant enzymes are needed to prevent oxidative stress and nerve damage.

In recent years, studies have shown that multiple nanozymes have endogenous antioxidant activity, such as SOD- and CAT-like activity, and can effectively protect nerve cells from oxidative damage. Therefore, nanozymes can mimic the endogenous antioxidant system and play a role in cell protection. There are hundreds of materials that can be used as nanozymes. For example, platinum nanoparticles, as nanozymes with CAT-like activity, can effectively alleviate ROS-induced oxidative damage. Therefore, the selection of nanomaterials with catalytic activity is the first step to prepare nanozymes with therapeutic effects on neurological diseases. In addition, the catalytic activities of nanozymes can be significantly improved by modifying some of their characteristics.

#### Changes in valence state promote catalytic activity

**Figure 2:**
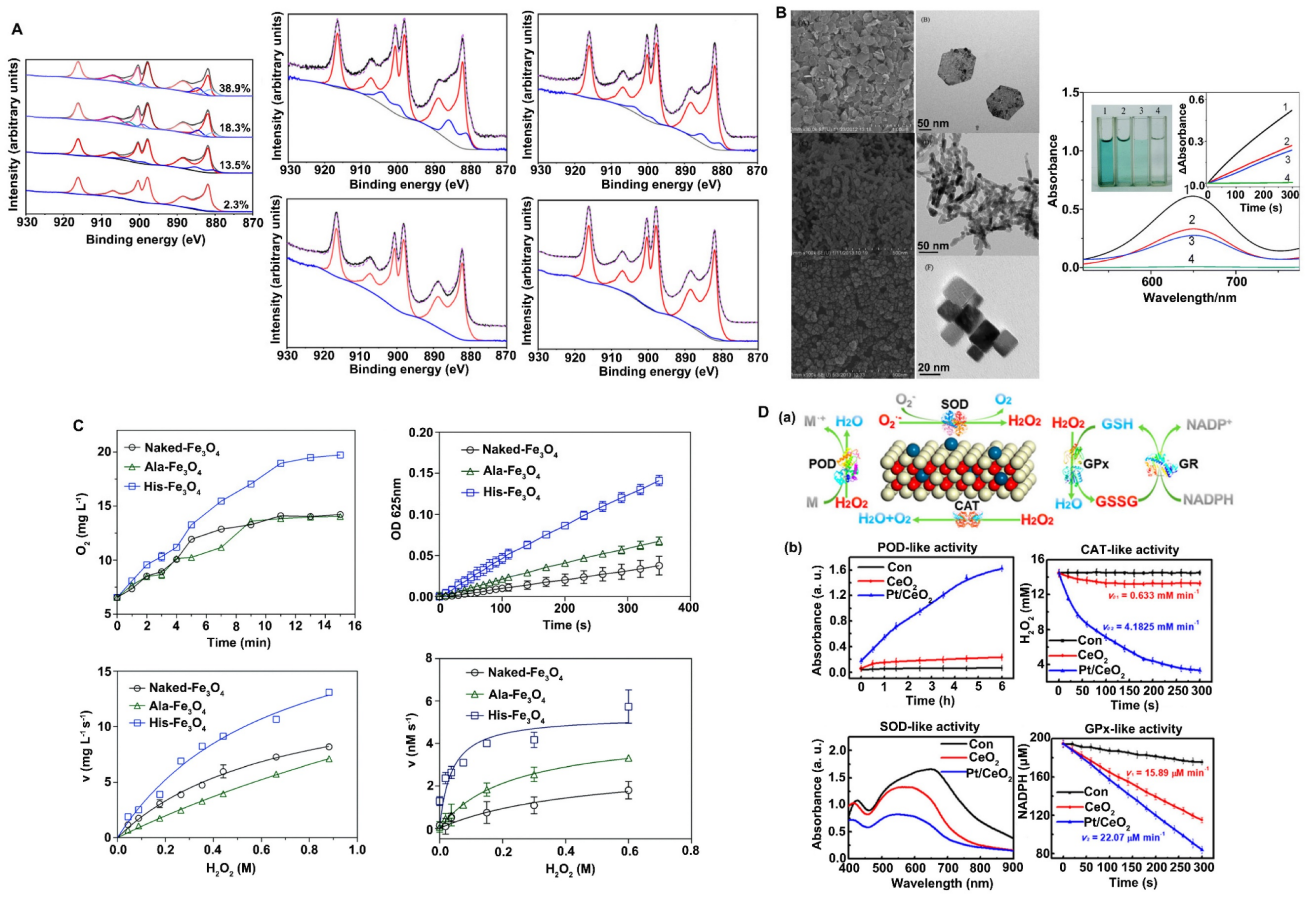
Strategies to improve the antioxidant capacities of nanozymes. A. The change of valence state promotes catalytic activities. The contents ratio of Ce^3+^ and Ce^4+^ on CeO_2_ surface affected CAT-like activity and SOD-like activity. Adapted with permission from 11, copyright 2019, IOP Publishing Ltd. B. Crystal plane regulation promotes catalytic activities. Morphology and the peroxidase-like activity of Co_3_O_4_ nanomaterials. Adapted with permission from 63, copyright 2014, Royal Society of Chemistry. C. Surface modification promotes catalytic activities. The histidine modification significantly improved the peroxidase-like and catalase-like activity of the Fe_3_O_4_ nanozymes. Adapted with permission from 66, copyright 2017, Royal Society of Chemistry. D. Metal doping modification promotes catalytic activities. POD-, CAT-, SOD-, and GPx-like activities with and without single-atom Pt/CeO_2_ catalysis. Adapted with permission from 56, copyright 2019, American Chemical Society.

The surface valence of nanozymes is closely related to their catalytic activities, and alterations in valence state and the proportion of atoms in each valence state lead to changes in catalytic activity and catalytic type. For example, Nelso et al. proposed that the ratio of Ce^3+^ and Ce^4+^ on the CeO_2_ surface has a great influence on the CAT-like and SOD-like activity (Figure 2A) 11. When the content of Ce^3+^ on the CeO_2_ surface is 40-60%, the nanozyme tends to exhibit SOD-like activity. However, when the content of Ce^4+^ is 70-80%, its CAT-like activity is more dominant. In addition, since the mechanism of the POD-like catalytic activity of CeO_2_ nanozymes is the Fenton reaction, similar to that of Fe_3_O_4_, the Ce^3+^/Ce^4+^ cyclic conversion rate determines the extent of this activity 62. A faster valence cycle means that H_2_O_2_ decomposition to ·OH can be catalyzed more quickly to achieve efficient oxidation of the substrate. In general, the higher the content of atoms in a low valence state on the surface is, the better nanozymes with Fenton catalytic properties can promote the forward reaction under acidic reaction conditions; that is, a higher content of atoms in a low valence state on the surface increases the decomposition rate of H_2_O_2_ and leads to stronger POD-like activity.

#### Crystal plane regulation promotes catalytic activity

Controlling crystal surface exposure is also a very effective strategy to regulate catalytic activity. In general, altering material morphology is a common strategy for crystal plane control, and different morphologies often lead to the exposure of different crystal faces. For different crystal planes, the arrangement of atoms and the number of dangling bonds are different, which can further affect the rate and selectivity of the enzyme-catalyzed reaction of materials. For example, Mu et al. prepared three crystal planes of Co_3_O_4,_ including {112}-nanoplates, {110}-nanorods and {100}-nanocubes (Figure 2B) 63. They found that the POD-like activity of Co_3_O_4_ nanomaterials is dependent on the crystal plane and that {112}-nanoplates exhibit better catalytic activity. The surface atoms of the three crystal planes are arranged in different ways and exhibit different electron transfer abilities, leading to different POD-like catalytic activities. In addition, Ge et al. prepared {100}-faceted palladium (Pd) nanocubes and {111}-faceted Pd octahedrons 64. They reported that {111}-faceted Pd octahedrons, which have lower surface energy, exhibit greater antioxidant enzyme-like activity and ROS-scavenging ability than {100}-faceted Pd nanocubes with higher surface energy. The results showed that the dissolution rate of O_2_ increased faster in the presence of Pd octahedrons than in the presence of Pd nanocubes, indicating that Pd octahedrons have higher catalase activity than Pd nanocubes. The turnover of O_2_^·-^ to O_2_ of Pd octahedrons is 7-fold higher than that of Pd nanocubes, indicating that Pd octahedrons have higher SOD-like activity.

#### Surface modification promotes catalytic activity

Since the catalytic reaction between nanozymes and substrates generally occurs on the surface of materials, the surface charge, active site exposure, substrate-binding ability and other properties of nanomaterials can be regulated by modifying the surface of the materials. Modification types include group modification, ion modification, and polymer modification. Surface modification can allow nanozymes to exhibit other types of catalytic activity, improve the catalytic specificity of enzymes, or inhibit a certain catalytic activity. Therefore, surface modification is a very flexible and controllable strategy to regulate the catalytic activity of nanozymes. For example, inspired by the ability of His-42 to enhance the adsorption of H_2_O_2_ through hydrogen bonding, Fan et al. introduced histidine onto the surface of Fe_3_O_4_ to improve its catalytic activity in decomposing H_2_O_2_ (Figure 2C) 65, 66. They prepared unmodified Fe_3_O_4_ nanozymes (Naked-Fe_3_O_4_), Fe_3_O_4_ nanozymes with alanine modification (Ala-Fe_3_O_4_) and Fe_3_O_4_ nanozymes with histidine modification (His-Fe_3_O_4_) and showed that the morphology and structure of the Fe_3_O_4_ nanozymes were not affected by amino acid modification. However, the catalytic activity of Fe_3_O_4_ nanozymes modified by His was significantly increased, while the catalytic activity of Ala-Fe_3_O_4_ was slightly increased. Moreover, modification with His improved the catalytic specificity of Fe_3_O_4_ and significantly enhanced the catalytic activity of Fe_3_O_4_ nanozymes in decomposing H_2_O_2_.

#### Metal doping modification promotes catalytic activity

For many catalytic reactions, a single metal-based catalyst cannot achieve both high selectivity and high catalytic activity. However, the synergistic action of different metals can improve the structure of nanozymes so that polymetallic nanozymes exhibit greater catalytic activities. For example, Wang et al. developed MnO_2_@PtCo nanoflowers and obtained multifunctional nanozymes with excellent catalytic efficiency by adjusting the ratio of reactants, where PtCo has OXD-like activity and MnO_2_ has CAT-like activity 67. In addition, Zhang et al. designed a Pt/CeO_2_ nanozyme with lasting catalytic activity. The Pt/CeO_2_ nanozyme is based on the principle of single-atom catalysis. The distribution of single-atom Pt led to the lattice expansion of the CeO_2_ nanozyme, which formed a new stable active site and electron transfer path, leading to a significant increase in its catalytic capacity (Figure 2D) 56. The Zhang group also reported PtPdMo trimetallic (PtPdMo triM) nanozymes with high catalytic activities and environmental selectivity. Nanozymes have homogeneous hollow cubic nanostructures with high catalytic activities and good selectivity. Due to lattice deformation and the high exposure of active sites, the catalytic activities of SOD and CAT were significantly improved 55.

### Improvement of the BBB-penetrating ability of nanozymes

The BBB is a physiological barrier between the blood and the central nervous system (CNS) that is mainly composed of brain capillary endothelial cells (BCECs) connected by tight junctions (TJs). The BBB is considered to be the most important safeguard against the passage of molecules and foreign substances through the extensive capillary network to the brain parenchyma. In addition to BCECs, the extracellular basement membrane, adjacent pericytes, astrocytes and microglia are all indispensable components of the BBB, which, together with the surrounding neurons, form a complex functional neurovascular system. The BBB can protect the relative stability of the internal environment of the CNS and prevent damage by harmful substances in the blood, which is highly important for the maintenance of CNS function and the CNS environment. However, for neurological diseases such as AD and PD, the BBB is a major obstacle to drug treatment. In general, most drugs cannot cross the BBB. Only small molecules with high lipid solubility and a relative molecular mass of < 400 can cross the barrier. To facilitate BBB transport, nanoparticles can be modified with targeting moieties so that they preferentially bind to receptors expressed at the BBB. In recent years, the development of nanodelivery technology has provided a series of strategies for the targeted delivery of drugs to the brain. For example, nanodelivery carriers such as liposomes, microemulsions, and micelles, modifiers such as cell transmembrane peptides (CPPs), and membrane transporters such as transferrin and lactoferrin have effectively improved the ability of drugs to reach brain tissue.

#### Lipid-coating modification

##### Polysorbate modification

**Figure 3:**
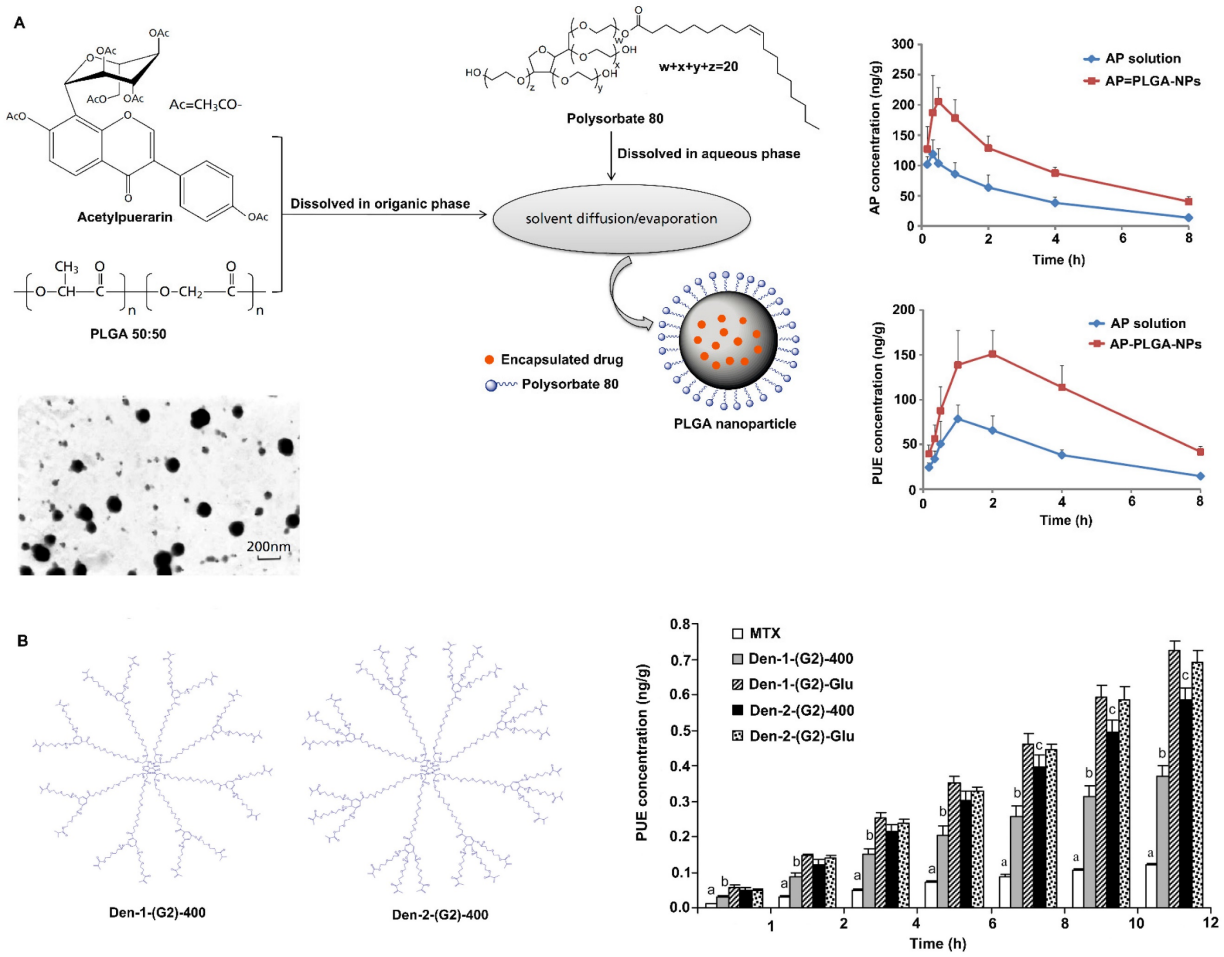
Strategies to improve the blood-brain barrier penetration of nanozymes by lipid-coating modification. A. Polysorbate 80 modification increases acetylpuerarin absorption in the brain. Adapted with permission from 74, copyright 2015, Oxford University Press. B. Polyether-copolyester (PEPE) dendrimers modification promote methotrexate across BBB. Adapted with permission from 81, copyright 2008, American Chemical Society.

Polysorbate-80 is a nonionic surfactant commercially known as Tween-80. Coating nanoparticles with polysorbate-80 has been shown to enhance drug delivery across the BBB. Studies have shown that nanoparticles coated with polysorbate-80 are similar to low-density lipoprotein (LDL) particles, which can absorb apolipoprotein E (ApoE) from blood plasma onto the nanoparticle surface and interact with LDL receptors, causing them to be taken up by endothelial cells, where they are then released and spread into the brain 68, 69. In addition, coating nanoparticles with polysorbate-80 inhibits P-glycoprotein on the BBB and inhibits the efflux system, thereby promoting BBB permeability 68, 70. Ramge et al. demonstrated that approximately 20-fold more polysorbate-80-coated nanoparticles were taken up by brain endothelial cells than uncoated nanoparticles 71. Moreover, intravenously or intranasally delivered 1% polysorbate-80-coated nanoparticles promoted higher drug distribution in the brain than uncoated nanoparticles or free drug 72, 73. Sun et al. reported that polysorbate-80-coated poly(lactic-co-glycolic acid) (PLGA) nanoparticles can improve the ability of acetylpuerarin (AP) to cross the BBB and enhance its brain-protective effects against cerebral I/R injury in rats (Figure 3A) 74. Chintamaneni et al. used polysorbate-80 surface-modified stearylamine (SA)-BQCA conjugated nanoparticles to treat AD. A study showed that coating with polysorbate-80 helped significantly improve the brain bioavailability of nanoparticles, allowing the coated nanoparticles to obviously prevent streptozotocin (STZ)-induced changes in memory, neuronal Aβ_1-42_, p-Tau, amyloid precursor protein (APP), nuclear factor-κB (NF-κB), and APP cleaving enzyme (BACE) levels and neuronal cell death 75.

##### Dendrimer surface modification

Dendrimers are customizable nanopolymers with uniform and well-defined particle sizes and shapes. Examples of dendrimers include poly(amidoamine) (PAMAM), poly(propylene imine) (PPI) and polyether-copolyester (PEPE), and Glyco and PEGylated dendrimers are very important 76-78. These dendrimers are able to carry drug molecules through physical interactions (encapsulation) or chemical bonding (prodrug approach), making them potential carriers for targeted drug delivery across the BBB. Surface-modified nanoparticles of PEPE dendrimers can cross the BBB and enter the brain by clathrin- and caveolin-mediated endocytosis 79, 80. In addition, PEPE dendrimers significantly improve the BBB-penetrating ability of nanoparticles without affecting the TJs between cerebral vascular endothelial cells after endocytosis 80. In addition, Dhanikula et al. reported that PEPE dendrimers can be used as drug carriers to effectively target and treat gliomas (Figure 3B) 81. The amount of methotrexate, an antitumor drug, transported across the BBB was found to be increased by three to five times after it was loaded in PEPE dendrimers. Furthermore, compared with nonglucosylated dendrimers, PEPE dendrimers conjugated to D-glucosamine enhance BBB permeability and tumor targeting, thus providing more effective treatment for glioma 81.

##### Modification by cell membrane coating

Cell membranes are natural materials in living organisms, conferring the advantage of biocompatibility. Compared with those modified by artificial membranes, nanoparticles modified by cell membranes have great advantages in the diagnosis and treatment of diseases due to the unique proteins, peptides and enzymes on the surface of the cell membranes. Nanoparticles modified with cell membranes have unique properties, such as extended blood circulation time, improved active targeting and enhanced cell internalization. In recent years, it has been found that blood cells such as red blood cells, white blood cells and platelet-modified nanozymes can circulate in the body for a long time and have certain targeting abilities.

**Figure 4:**
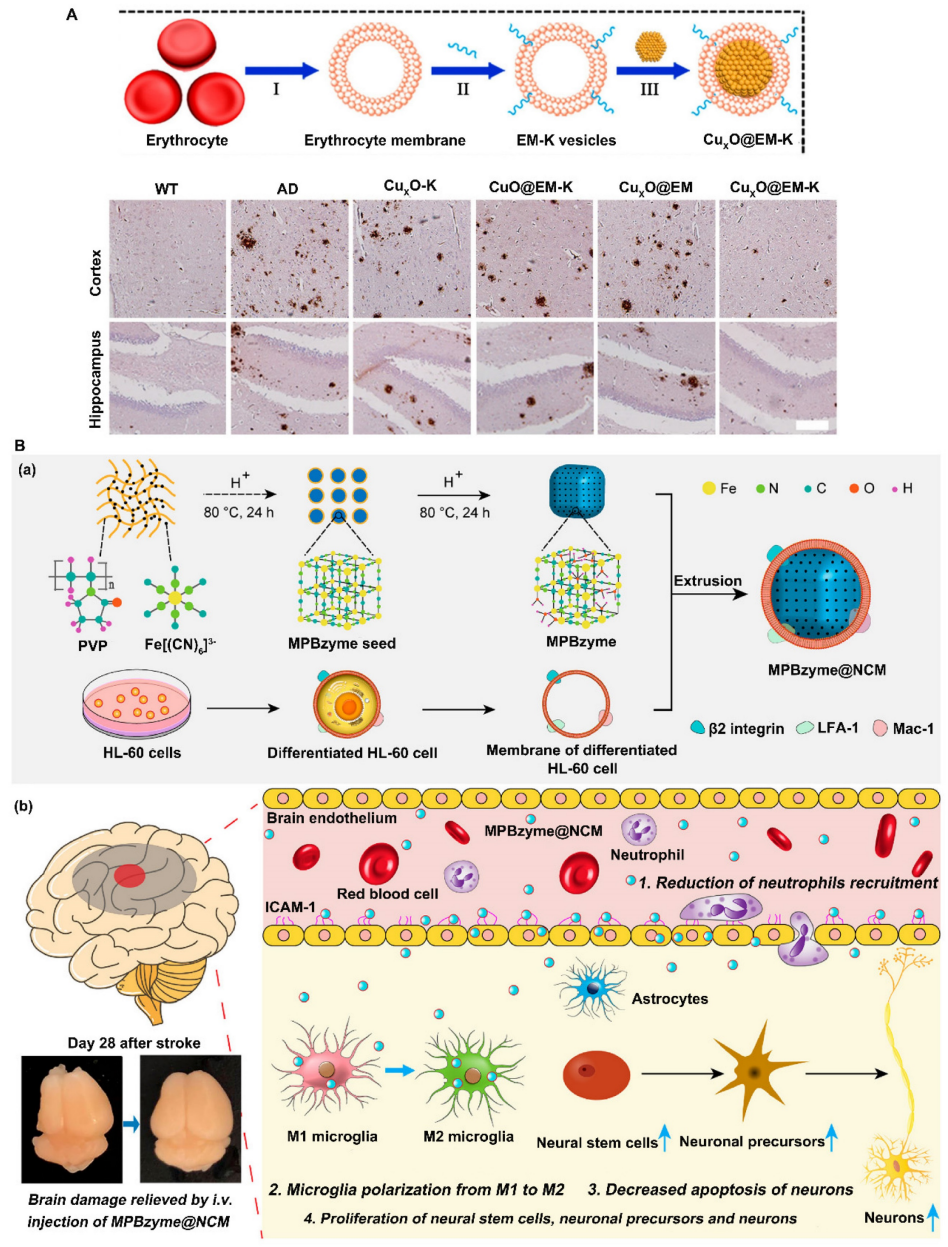
Strategies to improve the blood-brain barrier penetration of nanozymes by cell membrane coating. A. Erythrocyte membrane coated Cu*x*O@EM-K nanozymes increased stability and BBB permeability, and significantly reduced Aβ deposits. Adapted with permission from 49, copyright 2020, American Chemical Society. B. Neutrophil-like cell-membrane (NCM)-coated nanozyme (MPBzyme@NCM) therapy for ischemic stroke. Coating of NCM efficiently enhanced the penetration of nanozyme across brain microvascular endothelial cells. Adapted with permission from 39, copyright 2021, American Chemical Society.

Red blood cells (RBCs) were the first kind of cells to be used for cell membrane-based coating technology. RBCs are carriers that can circulate for a long time. Integrin-associated protein CD47 on the surface of RBCs interacts with signal regulatory proteins expressed by macrophages and dendritic cells, thereby enabling RBCs to evade immune surveillance and thus prolonging their blood circulation time 82, 83. RBCs also have a relatively large surface area and can carry large amounts of drugs or nanoparticles 84. Therefore, RBC membranes are the first choice for modifying nanoparticles. For instance, coating the surface of Cu*x*O nanozymes (Cu*x*O@EM-K) with 3xTg-AD mouse-derived erythrocyte membranes modified with the Aβ-targeting peptide KLVFF increased the stability and blood circulation time of the nanozymes, allowing them to capture more peripheral Aβ protein and transport it to the liver for degradation. KLVFF acts as a ligand to target Aβ and selectively captures Aβ in the blood in coordination with erythrocyte membranes. Moreover, coating with erythrocyte membranes prevents the formation of a protein corona, thus boosting the ability of the nanozymes to target Aβ (Figure 4A) 49.

Neutrophils are a type of white blood cell. Most white blood cells, including neutrophils, exhibit deformability that allows them to move easily from blood vessels to extravascular tissues. Therefore, nanoparticles wrapped by neutrophil membranes not only evade recognition by the immune system and cross physiological barriers but also have a targeting function through interactions with cell membranes 85. For example, Feng et al. reported that a neutrophil-like cell membrane-coated mesoporous Prussian blue nanozyme (MPBzyme@NCM) can promote the efficient delivery of nanozymes to damaged brain tissue after ischemic stroke due to the innate connections between microvascular endothelial cells and neutrophils (Figure 4B) 39. Experimental results have shown that MPBzyme@NCM nanozymes effectively target brain lesions and remove excess ROS to relieve oxidative stress. In addition, nanozymes promote microglial polarization to the M2 phenotype to exert anti-inflammatory effects, thus promoting neural recovery. Therefore, the integration of cell membrane technologies with nanozymes can lead to the generation of multifunctional nanozymes with desirable properties and enhance the biological application of nanozymes.

#### Protein-based modification

##### Lactoferrin-based modification

**Figure 5:**
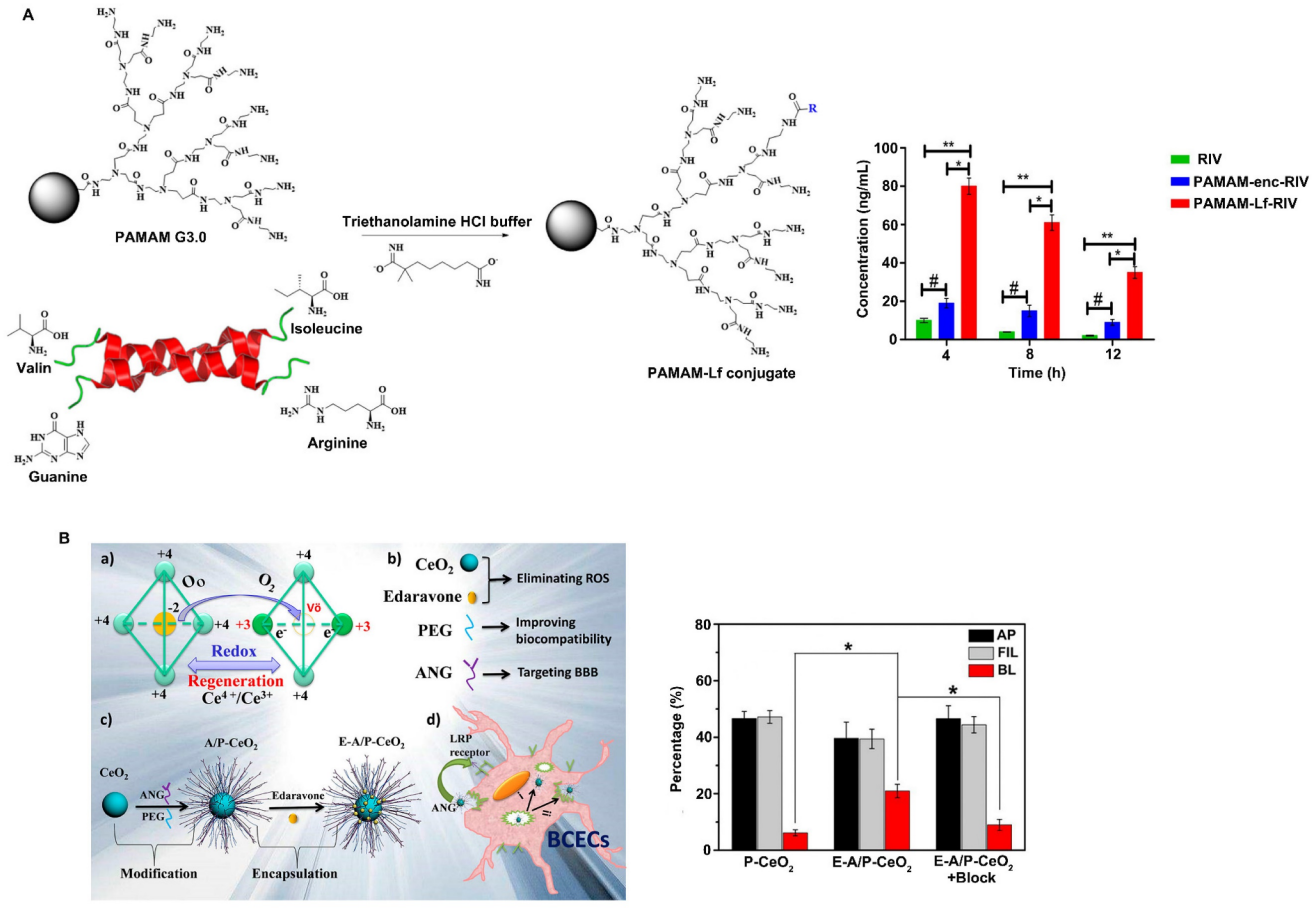
Strategies to improve the blood-brain barrier penetration of nanozymes by protein-based modification. A. Lactoferrin modified nanoparticles improved the brain delivery of rivastigmine (RIV). Adapted with permission from 89, copyright 2018, American Chemical Society. B. E-A/P-CeO_2_ nanoparticles modified with angiopep-2 (ANG-2) can rapidly cross the BBB and reach the cerebral infarction site. Adapted with permission from 92, copyright 2018, American Chemical Society.

Lf, also known as lactotransferrin, is a single-chain iron-binding glycoprotein. Lf is a nonspherical single chain in which the N-terminus and C-terminus are folded into two blade-like regions, the N-ring and C-ring, respectively. The two rings are connected by an α-helix, and there are two iron binding sites in the molecule, which can reversibly bind iron ions in the environment. Lf is a novel carrier for helping drugs cross the BBB 86. There are a large number of Lf receptors (LfR) on the cerebrovascular endothelial cells that comprise the BBB, and these receptors can bind specifically to Lf and mediate Lf transport into brain tissue through endocytosis 87. Therefore, Lf has great potential as a drug carrier for the targeted delivery of drugs into the brain. Therefore, an increasing number of studies have investigated the ability of nanoparticles surface-modified with Lf or nanocarriers loaded with Lf to achieve targeted drug delivery. For example, Wu et al. reported that surface modification with Lf significantly increased the efficiency of tanshinone I nanoemulsions in penetrating the BBB, indicating that Lf-modified nanoparticles have great potential for improving the brain delivery of drugs 88. Gothwal et al. conjugated Lf to polyamidoamine dendrimers to effectively deliver rivastigmine (RIV) to the brain (Figure 5A) 89. The study showed that Lf modification increased the bioavailability of RIV by 7.87-fold and significantly enhanced the uptake of RIV in the brain by 8-fold, ultimately improving the learning and memory of rats. In addition, Li et al. constructed Lf-Au-Bi_2_Se_3_ nanozymes for PD treatment. The results showed that the nanozymes could effectively reduce ROS levels, and the concentration of Lf-modified nanozymes in the brain was 2.67 times higher than that of unmodified nanoparticles. Moreover, due to the excellent targeting ability of Lf, Lf-Au-Bi_2_Se_3_ nanozymes are distributed near the mitochondria of cells, providing sustained protection of mitochondria 42.

##### Angiopep-2-based modification

Angiopeps are small peptides with 20 amino acids that can target the low-density lipoprotein receptor-related protein (LRP) domain and have been proven to have a good ability to assist vectors in penetrating the BBB. Angiopep-2 (ANG-2) exhibited greater BBB-penetrating ability than other LRPs or proteins with BBB-targeting capabilities, such as aprotinin and transferrin. ANG-2 crosses the BBB through LRP1-mediated endocytosis by specifically binding to LRP1 expressed on brain capillary endothelial cells. Studies have shown that the surface modification of nanoparticles with ANG-2 can effectively improve the BBB-penetrating ability of nanoparticles. For example, Huang et al. reported that an angiopep-conjugated dendrigraft poly-L-lysine (DGL)-based gene delivery system enhanced the cellular uptake of nanoparticles and increased the expression of genes loaded in nanoparticles in brain cells, ultimately improving the functional recovery of rotenone-induced PD model rats due to increased expression of GDNF in the brain 90. Zhong et al. loaded Prussian blue (PB) and ANG-2 onto the PAMAM surface to synthesize PPA NPs 91. The results showed that ANG-2-modified PPA nanoparticles exhibit excellent BBB-penetrating ability, as well as the ability to scavenge ROS and restore mitochondrial function in microglial cells. Furthermore, PPA nanoparticles can effectively reduce the accumulation of neurotoxic Aβ and restore the cognitive function of APP/PS1 model mice. Bao et al. designed ultrasmall ceria nanoparticles for the treatment of stroke and found that they exhibited improved targeting efficiency due to the presence of the targeting ligand ANG-2 on the surface (Figure 5B) 92. ANG-2-modified ceria nanoparticles cross the BBB to reach the cerebral infarction site through receptor-mediated endocytosis. Unlike unmodified P-CeO_2_ nanoparticles, E-A/P-CeO_2_ nanoparticles modified with ANG-2 can rapidly cross the BBB and accumulate in the brain, enhancing their antioxidant effect on brain tissue and significantly reducing the volume of damaged brain tissue. In addition, E-A/P-CeO_2_ nanozymes loaded with edaravone can effectively remove ROS and repair the damaged BBB, leading to enhanced alleviation of stroke.

### Inhibition of misfolded protein aggregation

#### Nanozymes modified by the KLVFF peptide inhibit Aβ aggregation in AD

**Figure 6:**
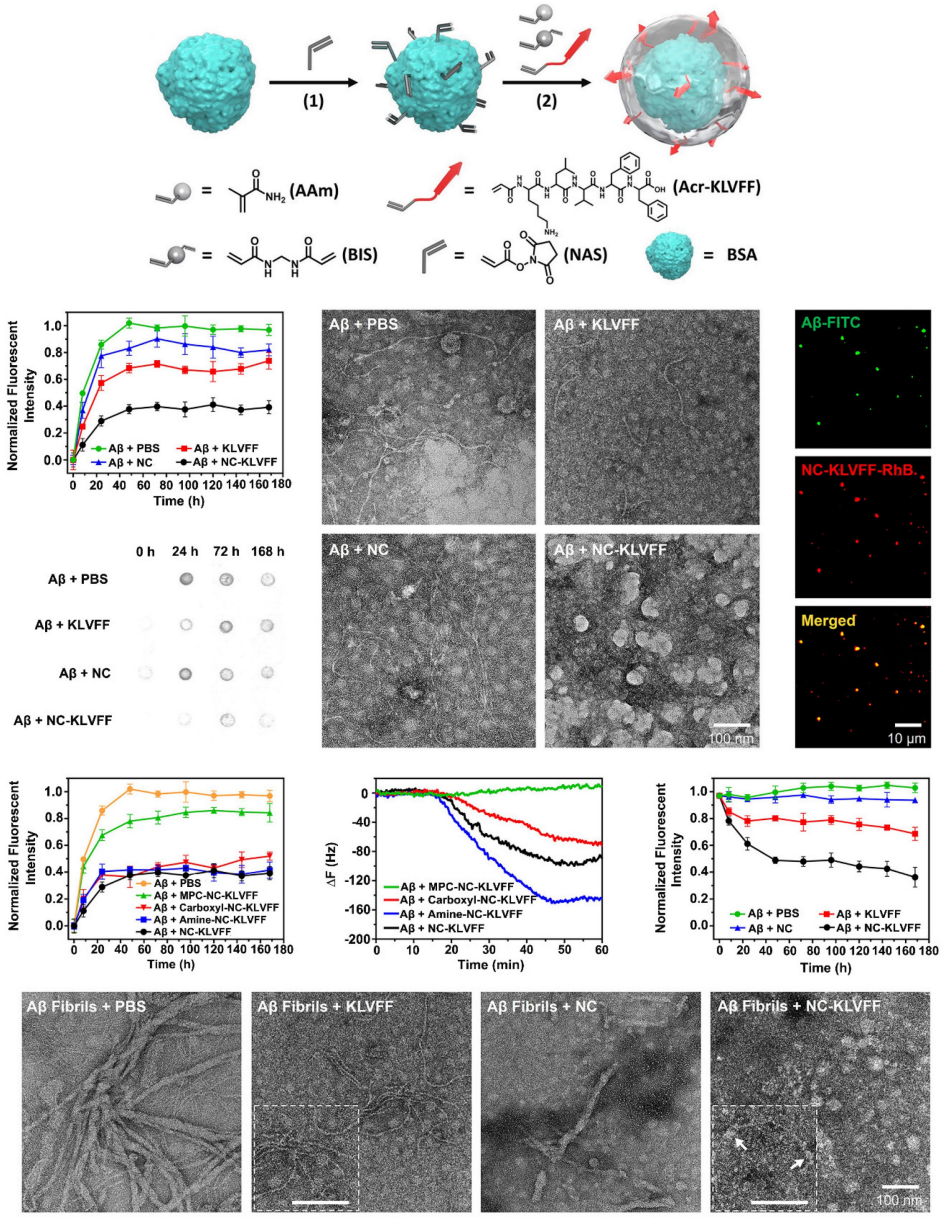
Strategies to inhibit the aggregation of misfolded proteins by modified KLVFF peptide. Nanozymes modified by KLVFF peptide inhibit Aβ aggregation and degrade Aβ fibril. Adapted with permission from 96, copyright 2019, American Chemical Society.

The peptide sequence KLVFF, derived from the Aβ42 sequence, effectively stabilizes soluble Aβ conformations and destabilizes unstable conformations, ultimately inhibiting Aβ aggregation. KLVFF is believed to be a major driver of Aβ fibrillation and can coassemble by strong H-bonding and hydrophobic interactions to target Aβ and inhibit its aggregation 93, 94. Previous studies have shown that KLVFF forms relatively large structures by covalently linking oligopeptides, dendritic macromolecules, or aliphicyclins, thereby enhancing the inhibition of Aβ aggregation through specific interactions and steric effects at the single-molecule level. Recently, some studies utilized the good affinity of KLVFF for Aβ and modified nanoparticles with KLVFF to capture and clear Aβ, thus reducing extracellular Aβ deposition 95. For example, Zhao et al. designed nanocomposites in which protein molecules were coated with polymer layers containing cross-linked KLVFF synthesized by in situ polymerization (Figure 6) 96. The nanocomposites inhibited the self-aggregation of Aβ and induced the dissociation of Aβ fibrils. Moreover, in vivo experiments demonstrated that the nanocomposites reduced the adhesion of Aβ aggregates to the neuronal surface, alleviated Aβ-induced neuronal injury, and restored the ability of endocranial microglia to phagocytose Aβ, ultimately protecting hippocampal neurons from apoptosis; thus, these nanocomposites represent a promising treatment for AD.

Nanozymes modified with the KLVFF peptide not only reduce the production of ROS but also significantly inhibit the aggregation of Aβ and extracellular Aβ deposition. For example, Ge et al. designed KLVFF@Au-CeO_2_ nanozymes in which the KLVFF peptide was loaded on the middle surface of Au NRs 51. The introduction of KLVFF peptide on the surface of nanozymes can lead to significantly increased decomposition of Aβ fibrils and inhibition of Aβ monomer aggregation, especially when combined with NIR, ultimately inhibiting Aβ-induced cytotoxicity in AD mice. Similarly, Ma et al. demonstrated that KD8@N-MCNs can cross the BBB by an Aβ transport-mimicking pathway and reduce the brain Aβ burden in 3xTg-AD model mice due to the presence of the KLVFF peptide on their surface. KD8@N-MCNs were found to inhibit Aβ_42_ aggregation and promote Aβ42 fibril disassembly 48.

#### Hydrophobic modification inhibits amyloid fibrillation

**Figure 7:**
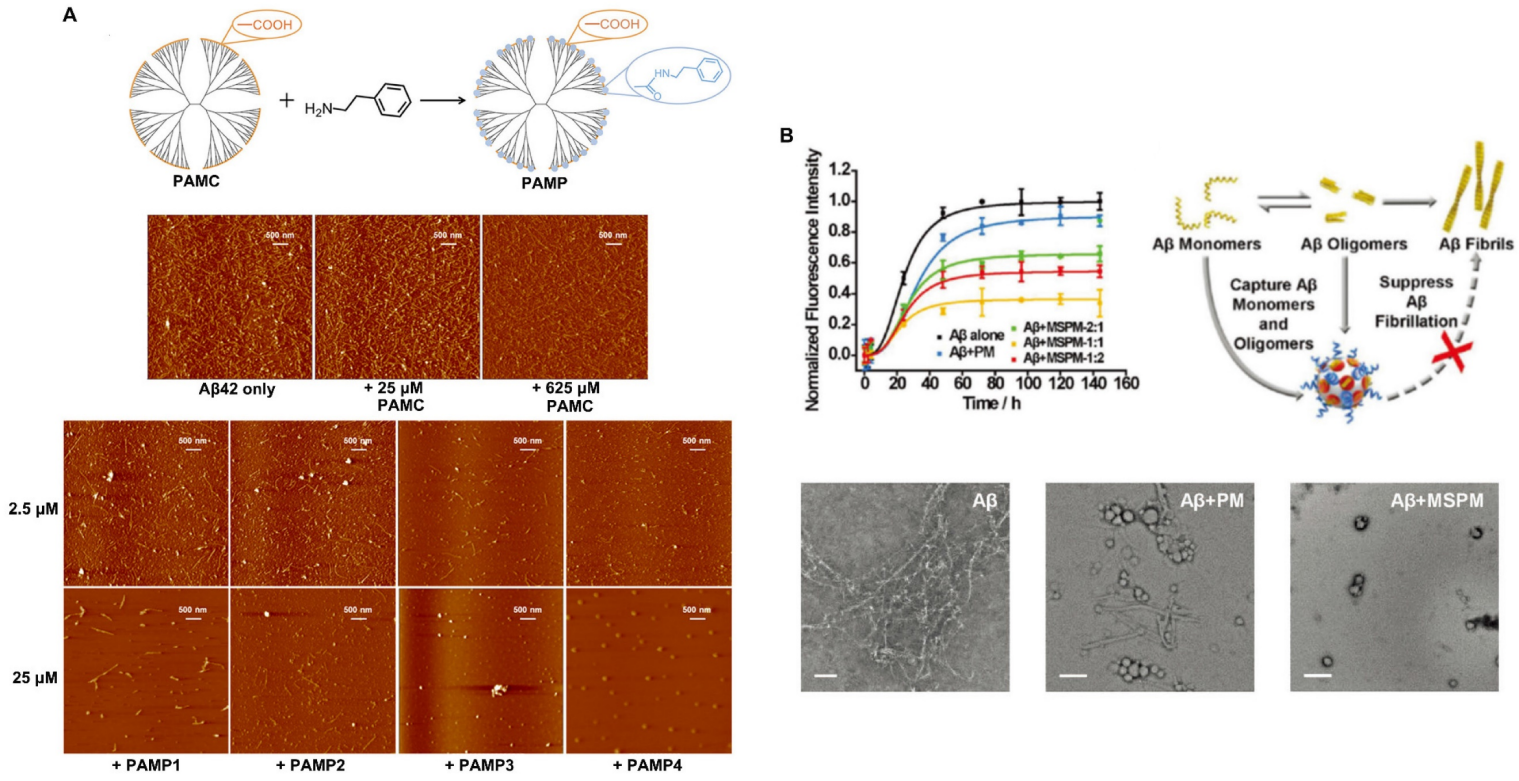
Strategies to inhibit the aggregation of misfolded proteins by hydrophobic modification. A. Phenylderivatized PAMC (PAMP) modification inhibiting Aβ aggregation through hydrophobic binding-electrostatic repulsion. Adapted with permission from 98, copyright 2018, American Chemical Society. B. The nanochaperone synthesized by self‐assembly of poly (β‐amino ester)‐block‐poly(ε‐caprolactone) (PAE‐b‐PCL) and poly(ethylene oxide)‐block‐poly(ε‐caprolactone) (PEG‐b‐PCL) can effectively recognize and inhibit Aβ aggregation. Adapted with permission from 99, copyright 2019, John Wiley and Sons.

Protein misfolding and the accumulation of highly ordered, insoluble aggregates rich in β-sheets are closely associated with a variety of NDDs, including AD, PD, HD and prion disease 97. Studies have shown that β-sheets are abundant in most cytotoxic aggregates. However, the cytotoxicity and pathogenicity of amyloid protein are mainly attributed to the aggregation of oligomer intermediates in the early stage of amyloid formation rather than to mature fibrils. Therefore, the inhibition of amyloid aggregation, especially of toxic β-sheet-rich intermediates, is an attractive therapeutic strategy for amyloid diseases. To this end, many nanomaterials with structures possessing hydrophobic recognition sites to capture Aβ peptides have been constructed. In addition, appropriate barriers are required to isolate Aβ peptides and inhibit Aβ aggregation. Nanomaterials also need to be able to promote Aβ transport to microglia and facilitate Aβ phagocytosis and clearance. A study showed that a synthesized phenylderivatized PAMC (PAMP) obtained by hydrophobic modification of the carboxyl terminus of a polyamidoamine dendrimer makes Aβ stretch into extended conformations that are distinct from β-sheet structures through hydrophobic binding-electrostatic repulsion, thus inhibiting Aβ aggregation (Figure 7A) 98. Carboxyl-terminated polyamidoamine (PAMC) is a biocompatible dendrimer with only carboxyl groups on its surface. After surface modification with phenethylamine (PEA) to introduce phenyl groups on PAMC, the surface contained only carboxyl groups and phenyl groups. Moreover, when the phenyl substitution degree was 30.5%, PAMP had the best performance, and the toxicity of Aβ aggregates was significantly reduced. In addition, inspired by a chaperone that can specifically recognize and adsorb hydrophobic fragments in abnormal proteins, a self-assembled nanochaperone based on a mixed-shell polymeric micelle (MSPM) was constructed. The nanochaperone was synthesized by the self‐assembly of poly (β‐amino ester)‐block‐poly(ε‐caprolactone) (PAE‐*b*‐PCL) and poly(ethylene oxide)‐block‐poly(ε‐caprolactone) (PEG‐*b*‐PCL). This nanochaperone has unique surface hydrophobic domains that recognize and bind to Aβ, while hydrophilic fragments can act as barriers to separate Aβ peptides from each other, allowing the nanochaperone to selectively capture Aβ peptides and effectively inhibit Aβ aggregation. Therefore, this nanochaperone can protect nerve cells against apoptosis and ameliorate cognitive deficits in AD mice by suppressing Aβ aggregation and reducing Aβ-induced cytotoxicity, as well as by promoting microglia-mediated Aβ elimination to alleviate Aβ-induced inflammation (Figure 7B) 99. Therefore, this modification may be a potential strategy for inhibiting Aβ aggregation for the treatment of AD.

## Conclusion and prospects

We have summarized the latest research progress and strategies for designing potential therapeutic nanozymes for the treatment of neurological diseases. Oxidative stress and neuroinflammation caused by oxidative damage are considered to be the major etiological factors of neurological diseases such as brain injury and NDDs. Cellular oxidative stress is induced by abnormally elevated ROS levels, which lead to DNA damage, inflammatory responses, and programmed cell death 100. Many nanozymes mimic endogenous antioxidant systems and can effectively protect nerve cells from oxidative damage by alleviating the oxidative stress induced by excess ROS. Although nanozymes address many shortcomings of traditional antioxidants, some nanozymes have lower catalytic activities and efficiency than natural enzymes and organic catalysts, so there is an urgent need to develop nanozymes with high catalytic activity for disease treatment. Based on the properties of nanozymes, it is possible to improve their antioxidant activity by adjusting their size, crystal morphology, or composition or by modifying their surface to enhance their therapeutic effect on ROS-related neurological diseases 101, 102. In addition, the balance among the multiple enzyme-mimicking activities of nanozymes needs to be studied. At present, nanozymes are mainly used to promote or inhibit ROS production for the treatment of various diseases. One kind of nanozyme usually has multiple enzyme activities 103. Importantly, the same nanozyme may even display different activities under different conditions. Balancing the antioxidase-like and peroxidase-like activities of nanozymes is of great importance to ensure that they exert effective and stable therapeutic effects. Accordingly, constructing environmentally responsive nanozymes or modifying nanozyme surfaces will be an effective measure to solve this problem. Although nanozymes show great potential application in the treatment of diseases, it is necessary to ensure the biosafety of nanozymes in vivo if they are to be applied in clinical practice in the future. In contrast to traditional proteases, nanozymes are mostly inorganic nanoparticles that can enter lysosomes, mitochondria or nuclei in cells and undergo irreversible interactions with intracellular components, thus causing damage to cells 104. In addition, as foreign substances, nanozymes may induce the body's immune response 105. To solve this problem, studies have shown that coating the surface of nanoparticles with polymers such as polyethylene glycol (PEG) and dextran can improve their biocompatibility. Therefore, enhancing the biocompatibility of nanozymes by surface modification or coating will be an effective strategy to improve the safety of nanozymes. The metabolism of nanozymes is a critical problem for clinical application. Some of the metal ions used to synthesize nanozymes are not essential to humans and organisms, and some metal components in nanozymes have the potential to induce inflammation and neurotoxicity. Some biodegradable organic nanozymes, such as polydopamine nanoparticles and melanin nanoparticles, are biodegradable and biocompatible. They can effectively remove RONS and subsequently be degraded into harmless substances and excreted out of the body, thus reducing toxicity and side effects in vivo.

In addition, nanomaterials composed of essential trace elements can be converted into active biological molecules in vivo to improve the bioavailability and biosafety of nanozymes 106. Nevertheless, the metabolism and biosafety of nanozymes will be key issues to be addressed for clinical application, and long-term clinical follow-up will be needed to understand the long-term effects 107.

Two major issues need to be solved before developing nanozymes to treat brain diseases. One aspect is to promote the effective crossing of the BBB by nanozymes. Currently, many nanozymes can penetrate the BBB, and certain modifications can effectively enhance their BBB permeability. The other aspect is that nanozymes need to target the damaged area of the brain. Although many nanozymes have good multiple enzyme-mimicking activities, their therapeutic effect is limited due to their inability to accurately target the damaged area of the brain. Although there are few relevant studies on the effective targeted treatment of brain injury by nanozymes, it has been shown that some specific peptides, such as cerebral stroke homing peptides, can be used to modify nanozymes to promote the targeting of nanozymes to brain injury tissue 40. Further development of more targeted peptides is needed. In the future, safer and more effective nanozymes are expected to be developed for the treatment of neurological diseases.

## Figures and Tables

**Table 1:** The potential application of nanozymes in neurological diseases.

Nanozymes	Functions	Type of diseases	Models	Therapeutic effects	References
Fe-N4	SOD and CAT activities	Ischemic stroke	middle cerebral artery occlusion (MCAO) model of rats	uric acid↓, ROS↓; Reduce infarct area; lipid peroxidation↓; alleviate neurological damage	33
HAS-Mn_3_O_4_	SOD activity	Ischemic stroke	Oxygen-Glucose Deprivation Model (OGD) induced cell model; MCAO model of mice	ROS↓; IL-1β↓, TNF-α↓, IL-6↓; SOD2↑; Inhibit autophagy activation, endoplasmic reticulum stress and brain tissue damage	34
Ce/Zeo-NMs	SOD and CAT activities	Ischemic stroke	middle cerebral artery occlusion-reperfusion (MCAO/R) model of rats	ROS↓; MMP2↑; MMP9↑; TNF-α↓, IL-6↓; Attenuated BBB disruption; Inhibit microglia and astrocytes activation	35
PEG-modified Fe_3_O_4_	SOD activity	Ischemic stroke	H_2_O_2_ and OGD induced PC12 cells model; MCAO model of mice	ROS↓; MDA↓; PECAM-1↑; ZO-1↑; Claudin-5↑; Protect BBB integrity; Reduces cerebral infarction and neuronal death	36
Co-Fe_3_O_4_	CAT activity	Ischemic stroke	MCAO model of mice	ROS↓; IL-1β↓, TNF-α↓, IL-6↓; Inhibit neuroinflammation; Reduce neurological deficits and decrease the infarct volume	37
Fe_2_NC@Se	SOD, CAT and GPx activities	Ischemic stroke	Oxygen and glucose deprivation/reoxygenation (OGD/R) induced cell model; MCAO model of rats	ROS↓; Improve neurological function, decrease brain infarct volumes and edema, ameliorate brain injury; Inhibit ASK1/JNK pathway	38
MPBzyme@NCM	SOD and CAT activities	Ischemic stroke	MCAO model of mice	ROS↓; promote microglia polarization toward M2, reduce the recruitment of neutrophils, and protect against neuronal damage; Recovery the long-term neurological function	39
PNzyme/MnO_2_	SOD and CAT activates	Ischemic stroke	MCAO model of mice	ROS↓; TNF-α↓; IL-6↓ Inhibit inflammation; reduces cerebral infarction; improve neurological function	40
Mnf	SOD, CAT and GPx activities	PD	1-methyl-4-phenylpyridinium (MPP+) induced PD-like cellular model	ROS↓; Inhibit caspases-3/7 activation	27
PBzyme	SOD and CAT activity	PD	1-methyl-4-phenyl-1,2,3,6-tetrahydropyridine (MPTP)-induced PD model of mice.	ROS↓; NLRP3 inflammasomes↓; caspase-1↓; GSDMD↓; reduces dopaminergic degeneration and inhibits neuroinflammation; alleviates motor deficits, attenuates the damage of mitochondrial membrane potential, and rescues dopaminergic neurons	41
Lf-Au-Bi_2_Se_3_	SOD, CAT and GPx activities	PD	MPTP-induced PD model of mice	ROS↓; Improve the memory and mobility, protect mitochondria, suppress dopaminergic neuron loss in the substantia nigra pars compacta	42
2D V_2_C MXenzyme	SOD, CAT and GPx activities	PD	MPTP-induced PD model of mice	ROS↓; tyrosine hydroxylase↑; IBA-1↓; Inhibit inflammation	43
S/Ce-PABMS	SOD and CAT activities	PD	MPTP-induced PD model of mice	ROS↓; IL-10↑; IL-1β↓; Inhibit inflammation; Decrease the α-synuclein aggregation; Inhibit microglia activation; Improved the spontaneous motor ability and motor coordination ability	44
Pd@PEG@Bor	SOD and CAT activities	AD	3×Tg-AD mice	ROS↓; Inhibit Aβ plaque deposition, alleviate neuron loss, and neuroinflammation; improve cognitive impairment	45
PEG-Fe_3_O_4_	SOD and CAT activities	AD	D-galactose induced aged mice	ROS↓; PECAM-1↑; Claudin5↑; ZO-1↑; improve neuroblast differentiation in the hippocampal dentate gyrus	46
TPP-MoS_2_ QDs	SOD and CAT activities	AD	APP/PS1 mice	ROS↓; IL-1β↓; IL-6↓; TNF-α↓; TGF-β↑; Degrade Aβ deposits; attenuate inflammatory	47
KD8@N-MCNs	SOD and CAT activities	AD	3×Tg-AD mice	ROS↓; IL-1β↓; TNF-α↓; Decrease Aβ deposits, ameliorate memory deficits, and alleviate neuroinflammation	48
Cu_x_O@EM-K	SOD and CAT activities	AD	3×Tg-AD mice	ROS↓; Reduce Aβ burden; ameliorate memory deficits	49
Nb_2_C MXenzyme	SOD and CAT activities	AD	APP/PS1 Transgenic mice	ROS↓; capture Cu^2+^; Decrease Aβ deposits; alleviate mitochondrial and neuroglial damage, suppress neuroinflammation; ameliorate cognitive deficits	50
KLVFF@Au-CeO_2_	SOD and CAT activities	AD	APP/PS1 Transgenic mice	ROS↓; Inhibit Aβ aggregation and degrade Aβ fibril; improve the cognitive function	51
BNPs	SOD activity	HD	mHTT deposits induced cell model	Capture Cu^2+^; Reduce mitochondria oxidative stress and disaggregate mHTT	53
carbogenic nanozyme	SOD activity	TBI	H_2_O_2_- or LPS-stimulated cell models; Chronic constriction injury induced TBI mice model	ROS↓; RNS↓; MMP-9↓; Improve BBB permeability; reduce brain edema; improve the spatial memory capacity	54
PtPdMo triM	SOD and CAT activities	TBI	LPS-induced mice model of brain injury	ROS↓; RNS↓; Suppress neuroinflammation; attenuate brain injury and improve memory	55
Pt/CeO_2_	SOD, CAT and GPx activities	TBI	LPS-induced cell model; establish TBI mice model with fluid percussion injury	ROS↓; RNS↓; Improve the wound healing of neurotrauma and reduce neuroinflammation	56
Cr/CeO_2_	SOD, CAT and GPx activities	TBI	establish TBI mice model with fluid percussion injury	ROS↓; RNS↓; MMP-9↓; Improve the wound healing and reduce neuroinflammation; improve neuronal cognition	57
O-NZ	SOD and GPx activities	TBI	establish TBI mice model with fluid percussion injury	ROS↓; RNS↓; Rescue long-term neurocognition; reduce acute neuroinflammation; activate Nrf2/HO-1 pathway	58
RhN_4_, VN_4_ and Fe-Cu-N_6_	CAT and GPx activities	TBI	establish TBI mice model with fluid percussion injury	Accelerate the scalp healing from brain trauma and reduce inflammation	59

## Abbreviations

4-HNE: 4-hydroxynonenal
ɑ-syn: alpha-synuclein
Aβ: amyloid-beta
AD: Alzheimer's disease
ANG-2: Angiopep-2
AP: acetylpuerarin
ApoE: apolipoprotein E
APP: amyloid precursor protein
BACE: APP cleaving enzyme
BBB: blood‒brain barrier
CAT: catalase
BCECs: brain capillary endothelial cells
CNS: central nervous system
CPPs: cell transmembrane peptides
GOx: glucose oxidase
GPx: glutathione peroxidase
GSDMD: gasdermin D
HAS: Human serum albumin
HD: Huntington's disease
HO-1: heme oxygenase-1
HRP: horseradish peroxidase
HTT: huntingtin
IL-1β: interleukin 1β
IL-6: interleukin 6
I/R: ischemia‒reperfusion
LDL: low-density lipoprotein
Lf: lactoferrin
LfR: Lf receptors
LRP: low-density lipoprotein receptor-related protein
MCAO/R: middle cerebral artery occlusion reperfusion
MDA: malondialdehyde
mitoROS: mitochondrial reactive oxygen species
mHTT: mutant HTT
NDDs: neurodegenerative diseases
NIR: near-infrared region
NF-κB: nuclear factor-κB
NLRP3: leucine-rich repeat family pyrin domain containing 3
Nrf2: nuclear factor erythroid-2 related factor 2
OGD/R: oxygen and glucose deprivation/reoxygenation
OXD: oxidase
PAE‐*b*‐PCL: poly (β‐amino ester)‐block‐poly(ε‐caprolactone)
PAMAM: poly(amidoamine)
PAMP: phenylderivatized PAMC
PARP: polyadenosine diphosphate ribose polymerase
PD: Parkinson's disease
PEA: phenethylamine
PECAM-1: platelet endothelial cell adhesion molecule-1
PEG: polyethylene glycol
PEG‐*b*‐PCL: poly(ethylene oxide)‐block‐poly(ε‐caprolactone)
PEPE: polyether-copolyester
PLGA: polysorbate-80-coated poly(lactic-co-glycolic acid)
POD: peroxidase
PPI: poly(propylene imine)
RBCs: red blood cells
redox: oxidation‒reduction
RNS: reactive nitrogen species
RONS: reactive oxygen and nitrogen species
ROS: reactive oxygen species
SOD: superoxide dismutase
TBI: traumatic brain injury
TGF-β: anti-inflammatory transforming growth factor β
TNF-α: tumor necrosis factor-α
TJs: tight junctions
TJPs: tight junction proteins
STZ: streptozotocin
ZO-1: Zonula Occluden-1
